# Multimodal data-driven eye-movement subtypes and their cerebral glucose metabolic patterns in Parkinson’s disease

**DOI:** 10.3389/fnagi.2026.1794652

**Published:** 2026-03-11

**Authors:** Yifan Zhang, Wenli Zhang, Guoyang Li, Jing Huang, Huahui Zou, Xucheng Zhang, Xiangcheng Wang, Xiaoguang Luo

**Affiliations:** 1 Department of Neurology, Shenzhen People’s Hospital, The Second Clinical Medical College of Jinan University, Shenzhen, China; 2 Department of Neurology, Shenzhen Baoan People’s Hospital, Shenzhen, China; 3 Clinical Research Center of Geriatric Medicine, Shenzhen People’s Hospital, The Second Clinical Medical College of Jinan University, Shenzhen, China; 4 Institute of Software, Chinese Academy of Sciences, Beijing, China; 5 Department of Nuclear Medicine, Shenzhen People’s Hospital, The Second Clinical Medical College of Jinan University, Shenzhen, China; 6 The First Affiliated Hospital of Southern University of Science and Technology, Shenzhen, China

**Keywords:** brain metabolism, eye tracking, FDG-PET, oculomotor subtypes, Parkinson’s disease

## Abstract

**Background:**

Previously reported Parkinson’s disease (PD) subtyping schemes often show limited stability and cross-cohort generalizability.

**Objective:**

To derive data-driven oculomotor subtypes in PD using multi-task eye-movement assessment and to characterize their cerebral glucose metabolic patterns.

**Methods:**

We administered a non-invasive multi-task eye-movement battery to 122 patients with PD and 69 healthy controls. Multidimensional oculomotor features were analyzed using unsupervised k-means clustering to identify PD subtypes. In a PD subset undergoing ^18^F-fluorodeoxyglucose positron emission tomography (FDG-PET; *n* = 30), regional cerebral glucose metabolism was quantified to compare metabolic profiles between subtypes.

**Results:**

Clustering identified two PD subtypes: an oculomotor-efficient subtype (PD-E) and an oculomotor-inefficient subtype (PD-I). The subtypes differed across multiple oculomotor parameters, with antisaccade (AS) metrics showing the most prominent divergence. Compared with PD-I, PD-E showed higher FDG uptake in frontotemporal cortices. Metabolic differences were directionally concordant with groupwise patterns in cognitive measures.

**Conclusion:**

Integrating eye-movement digital phenotypes with FDG-PET metabolism may provide complementary information for cognitive-domain profiling and assessment in PD. Longitudinal studies and independent cohort validation are needed to confirm stability and clinical translatability.

## Introduction

Parkinson’s disease (PD) is a progressive neurodegenerative disorder with marked heterogeneity, and is frequently accompanied by characteristic eye-movement abnormalities. Eye movements are naturally generated and objectively quantifiable measures. They can reflect dysfunction within PD-related neural circuits and may serve as sensitive indicators of early or prodromal changes. These abnormalities are thought to reflect dysfunction across the cortico–basal ganglia–superior colliculus–cerebellar network involved in oculomotor control (Leigh and Zee, 2015).

Core oculomotor paradigms include saccadic and smooth pursuit (SP) eye movements. Saccades are rapid, ballistic shifts of gaze that enable efficient redirection of fixation between targets. Their generation relies on spatial encoding within occipital and parietal cortices projecting to the superior colliculus, higher-order gating mediated by frontal cortex–basal ganglia circuits via the substantia nigra pars reticulata to the superior colliculus, motor execution through the superior colliculus–brainstem saccade generators, and cerebellar mechanisms that calibrate saccade amplitude and endpoint accuracy (Wang et al., 2025). Patients with PD commonly exhibit reduced saccade amplitude, decreased velocity, and fragmented trajectories, with impairments often more pronounced in the vertical plane (Shaikh and Ghasia, 2019). Upward visually guided saccadic gain has been reported to be reduced in PD and may provide supportive discrimination from healthy controls in specific paradigms, but it is sensitive to aging and is not a standalone diagnostic biomarker (Irving and Lillakas, 2019; Waldthaler et al., 2019). Antisaccade (AS) error rates are closely associated with executive dysfunction and global cognitive performance (Waldthaler et al., 2019, 2021). SP eye movements represent continuous, velocity-adjustable tracking of moving targets. In PD, pursuit gain and tracking velocity are frequently reduced and may correlate with disease severity (Frei, 2021).

In the differential diagnosis of parkinsonian syndromes, eye-movement analysis can capture distinct patterns of circuit-level dysfunction, including fronto–basal ganglia imbalance, cerebellar oculomotor abnormalities, and impairment of vertical saccadic pathways (Diotaiuti et al., 2025). These features may aid in differentiating PD-predominantly characterized by abnormalities in voluntary saccades and predictive SP-from multiple system atrophy, and progressive supranuclear palsy. In severe cases, gaze limitation (Kassavetis et al., 2022; Pinkhardt and Kassubek, 2011), as a potential digital biomarker for prodromal PD, eye tracking has been used to differentiate PD from healthy individuals and to support early identification of disease-related changes (Chudzik et al., 2024; Tsitsi et al., 2021). In addition, patients with PD may exhibit shortened fixation duration, increased susceptibility to distraction, and difficulty maintaining stable fixation, which have been associated with cognitive dysfunction (Tsitsi et al., 2021). Compared with conventional clinical scales, eye-movement measures are more objective and impose a lower cognitive burden. Accordingly, they are considered promising digital biomarkers for assessing cognitive status and disease progression in PD (Diotaiuti et al., 2025). Previous studies have shown that multi-paradigm eye-movement parameters are associated with PD stage, disease severity, and multiple cognitive domains (Koch et al., 2024), and may help distinguish cognitive subgroups within PD (Adams et al., 1993). In particular, saccadic amplitude, latency, and error rates may sensitively track the progressive transition from cognitively normal PD to PD dementia (Liao et al., 2024).

Although PD patients exhibit abnormalities across multiple oculomotor components, including saccade initiation, amplitude and velocity control, and SP (Antoniades and Spering, 2024). However, complex interactions among eye-movement measures hamper the diagnostic utility of single tasks or individual parameters. Data-driven and artificial intelligence (AI) approaches have therefore been applied to oculomotor data, revealing distinct subtypes of oculomotor impairment within PD that differ in cognitive performance (Waldthaler et al., 2023). Prior studies suggest that multimodal integration of eye-movement, magnetic resonance image (MRI), and gait features provides complementary information for predicting cognitive status in PD (Liang et al., 2025). Moreover, multi-domain eye-movement features can distinguish PD patients across levels of cognitive impairment and, and show preliminary utility in classifying PD with mild-to-moderate motor dysfunction (Brien et al., 2023; Koch et al., 2024). Longitudinal cohort studies further indicate that data-driven cognitive subtypes follow domain-specific trajectories of cognitive progression (Pourzinal et al., 2023).

Existing evidence further suggests that heterogeneity across study paradigms may constrain the generalizability of eye-movement metrics and complicate mechanistic interpretation. In contrast, ^18^F- fluorodeoxyglucose (FDG) positron emission tomography (PET) is a well-established modality for assessing cerebral glucose metabolism. FDG-PET–derived network patterns, including the PD–related pattern (PDRP) associated with motor severity and the PD cognitive pattern (PDCP) linked to cognitive impairment, have been extensively validated (Peralta et al., 2019). FDG-PET analyses have also shown good diagnostic performance in the differential diagnosis of PD (Sun et al., 2022; Zhao et al., 2024).

Moreover, a growing body of work has established machine-learning and AI-based approaches to further enhance PET-based discrimination (Wang et al., 2024; Zhao et al., 2024), and has integrated functional MRI to improve the characterization of disease-related network alterations (Li X. et al., 2025). Beyond diagnostic utility, PD-specific metabolic signatures and their associations with clinical phenotypes have been increasingly documented (Butterfield et al., 2022), and PET-derived motor-related network activity appears to evolve with disease progression (Ko et al., 2013). Importantly, distinct PD motor subtypes exhibit differential metabolic profiles, and patterns of hypometabolism have been linked to cognitive decline and longitudinal cognitive trajectories (Huang et al., 2007a).

Despite these advances, systematic integration and validation of eye-movement features with FDG-PET–derived network patterns remain insufficient. In this study, we identified distinct oculomotor functional subtypes within PD using high-dimensional multi-task eye-movement data. We then integrated these subtypes with ^18^F-FDG PET–derived cerebral glucose metabolic network patterns to explore their potential neural substrates and associations with clinical phenotypes.

## Materials and methods

### Study design and participants

This study adopted a single-center, cross-sectional design. Participants included patients with PD and age- and sex-matched healthy control (HC). A subset of PD patients additionally underwent ^18^F- FDG PET imaging to assess cerebral glucose metabolism. All eye-movement tests in PD patients were performed in the medication-off (OFF) state, following an overnight withdrawal of dopaminergic medications for at least 12 h prior to testing, in accordance with established clinical research standards (Thobois et al., 2004; Zacharia et al., 2023). Eye-movement testing and ^18^F-FDG PET imaging were scheduled within the same time window.

The diagnosis of PD was established according to the Movement Disorder Society (MDS) clinical diagnostic criteria (Postuma et al., 2015) by experienced neurologists. HCs were recruited contemporaneously from community volunteers or individuals undergoing routine health examinations and had no history of neurological or psychiatric disorders. Inclusion criteria were: age ≥ 18 years; normal or corrected-to-normal vision sufficient to complete eye-movement tasks; and the ability to complete all cognitive and emotional assessments and eye-movement testing. Exclusion criteria included: comorbid major neurological disorders; severe psychiatric illness or a history of substance or alcohol abuse; significant structural brain abnormalities that could affect the interpretation of ^18^F-FDG PET findings; and ocular or extraocular muscle disorders that might interfere with eye-movement measurements.

**FIGURE 1:**
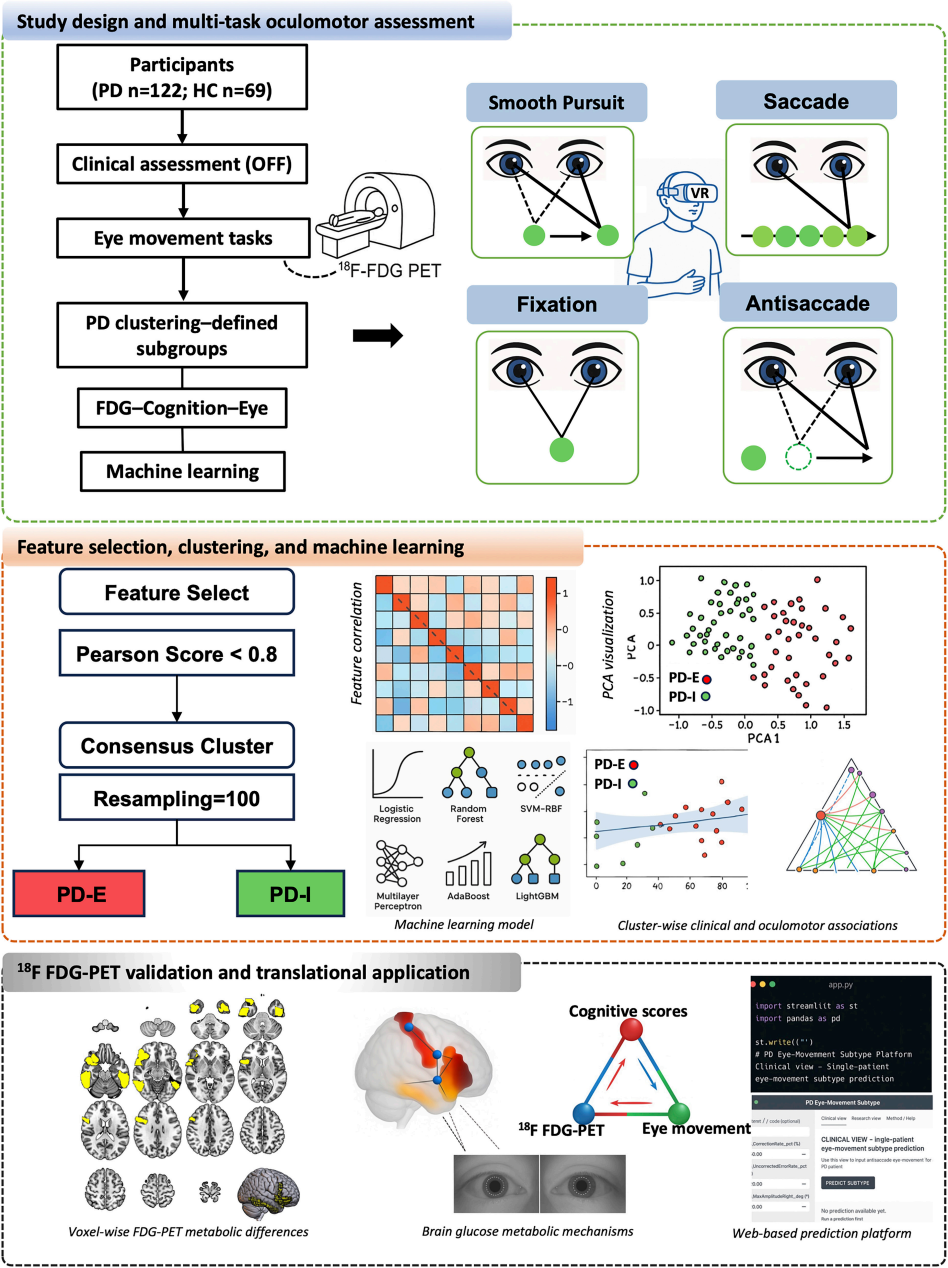
Study design, multi-task oculomotor assessment, and analytical workflow.

In total, 122 PD patients and 69 HCs were included. Owing to study constraints, only a subset of PD patients underwent ^18^F-FDG PET imaging, yielding an imaging subsample of 30 patients. A schematic of the study workflow is presented in Figure 1, and a video illustrating the oculomotor task is provided in Supplementary Video 1.

### Clinical motor and non-motor assessments

Demographic information was collected for all participants, including age, sex, body mass index, and years of education. For patients with PD, additional clinical data were recorded, including disease duration, Hoehn and Yahr (H&Y) stage, and levodopa equivalent daily dose (LEDD). Motor and non-motor symptoms were assessed using the Movement Disorder Society–sponsored revision of the Unified Parkinson’s Disease Rating Scale (MDS-UPDRS) Parts I–IV, supplemented by the Fatigue Severity Scale (FSS), Epworth Sleepiness Scale (ESS), and Scales for Outcomes in Parkinson’s Disease–Autonomic (SCOPA-AUT). Mini-Mental State Examination (MMSE) and the Montreal Cognitive Assessment (MoCA). Psychiatric symptoms and quality of life were assessed using the Hamilton Anxiety Scale (HAMA), Hamilton Depression Scale (HAMD), Geriatric Depression Scale (GDS), STAI, and the PDQ-39.

### Eye-movement tasks and feature extraction

Eye movements were recorded using the pupil center–corneal reflection method with the EyeKnow eye-tracking and analysis system (Beijing Zhongkeruiyi Information Technology Co., Ltd., Beijing, China) at a sampling rate of 120 Hz. Prior to testing, all participants underwent a standardized five-point calibration, and formal data acquisition proceeded only when the maximum calibration error was within a 2° visual-angle radius. All oculomotor parameters were automatically computed under unified settings using the system’s built-in processing modules. The experimental paradigms comprised three major categories: SP, saccadic eye movements, and fixation stability. In the SP (SP) tasks, a green target moved along a sinusoidal trajectory under three conditions: horizontal SP with a 20° amplitude (SP), horizontal smooth pursuit with a 15° amplitude (SP1), and vertical smooth pursuit with a 15° amplitude (SP2), all at a frequency of 0.2 Hz. Participants were instructed to track the target continuously and as accurately as possible. Extracted metrics included initiation time, tracking velocity and acceleration, number and magnitude of deviations (> 4°), eye-opening rate, overshoot and undershoot counts, pursuit gain, and overall tracking accuracy. Saccadic tasks included overlap saccade (OP) (central fixation presented for 1.0 s, with a peripheral target appearing at ± 15° horizontally or vertically while the central fixation remained for an additional 0.2 s), gap prosaccade (GP) (central fixation presented for 0.8 s, followed by a 0.2 s gap before target onset at ± 15°), and AS (stimulus presentation similar to prosaccade trials, but requiring a saccade to the mirror location opposite the target). For these tasks, accuracy, latency, fastest and mean reaction times, and average and peak saccadic velocities were quantified. Fixation stability was assessed using a lateral fixation (LF) holding task, in which participants sequentially fixated on a central target and on targets positioned at ± 15° to the left, right, up, and down, each held for 6 s; outcome measures included deviation counts (> 4° and > 2°), cumulative deviation, total deviation duration, and fixation accuracy. In addition to raw oculomotor parameters, standardized cognitive and motor function scores generated by the EyeKnow system were used as auxiliary derived indices. This pipeline performs unified resampling, linear interpolation, and normalization of high-frequency multivariate eye-movement trajectories (Butler et al., 2025), and extracts activity features, behavioral features, phase-error features related to pursuit gain, stability, and offset, as well as higher-order descriptors including trajectory complexity, fixation entropy, and time- and frequency-domain statistics (Opwonya et al., 2023; Rykov et al., 2024). Based on these features, a multitask “regression + classification” learning framework was applied to generate composite scores, combining a feedforward neural network for statistical features with deep learning models for time-series features, to comprehensively characterize functional states associated with oculomotor behavior (Jeong et al., 2022; Sakal et al., 2024). Demonstration videos of each eye-movement paradigm are provided in Supplementary Video 2.

### FDG-PET acquisition and processing

^18^F-FDG PET imaging was performed on an integrated PET/MR system (uPMR 790; United Imaging Healthcare, Shanghai, China). PET images were reconstructed using a regularized ordered-subset expectation maximization (ROSEM) algorithm. Post-processing of ^18^F-FDG PET images was performed in the MATLAB environment (MathWorks, Natick, MA, United States) using Statistical Parametric Mapping (SPM8) in combination with in-house scripts. For each participant, FDG-PET images were spatially coregistered to the corresponding T1-weighted (T1w) structural MRI using a rigid-body transformation with normalized mutual information as the cost function, ensuring accurate within-subject alignment between metabolic and anatomical images. The T1w images were subsequently segmented and non-linearly normalized to the Montreal Neurological Institute standard space in SPM8, and the resulting deformation parameters were applied to the corresponding FDG-PET images to achieve consistent spatial normalization in standard space. At the PET image level, voxel-wise standardized uptake values (SUVs) were computed from the reconstructed images. Standardized uptake value ratio (SUVR) maps were derived by normalizing SUV images to a reference region. In Montreal Neurological Institute space, regional SUVRs were extracted using the Automated Anatomical Labeling1 (AAL1) atlas (Antoniades et al., 2013). For cortical and subcortical regions, SUVRs were calculated using the whole cerebellum as the reference region (Wu et al., 2023), whereas for cerebellar regions, SUVRs were normalized to the voxel-wise global mean brain metabolism (Ren et al., 2025). Representative 2D slices were generated using MRIcroGL.

### Machine-learning–based prediction of PD oculomotor subtypes

Using 67 representative oculomotor features, supervised learning models were constructed to predict oculomotor subtypes, with clustering-derived labels serving as pseudo–ground truth. Model inputs were restricted to six AS task metrics—correction rate, uncorrected error rate, and maximal saccade amplitudes in the up, down, left, and right directions—to balance interpretability and dimensionality reduction. All feature selection and preprocessing steps were strictly confined to the training data to prevent information leakage. Continuous variables were standardized using z-score normalization within a unified modeling pipeline, with scaling parameters estimated exclusively from the training set and consistently applied to the test set and subsequent inference. Multiple binary classifiers were evaluated under a common framework, including logistic regression (L1/L2 regularization), random forest, radial basis function support vector machine (SVM-RBF), multilayer perceptron (MLP), AdaBoost, XGBoost, and LightGBM, using conservative parameter settings to balance model capacity and overfitting risk. Performance evaluation employed a stratified 75%/25% train–test split and five-fold stratified cross-validation within the training set. The primary endpoint was the area under the receiver operating characteristic curve (AUC) on the independent test set, with accuracy and F1 score as secondary metrics; concordance between cross-validated and test-set AUCs was examined to assess generalization stability. Following model selection, the final classifier was refit on the full dataset using identical preprocessing and parameter settings, and the complete pipeline (standardization and classifier) was serialized via joblib (.pkl) for deployment. An interactive web application was subsequently developed using Streamlit to enable single-case input and batch prediction, providing oculomotor-inefficient Parkinson’s disease subtype (PD-I)/oculomotor-efficient Parkinson’s disease subtype (PD-E) subtype outputs with probabilistic estimates and generating receiver operating characteristic (ROC) curves, confusion matrices, and classification reports.

### Statistical analysis

According to variable distributions, independent-samples *t*-tests, Mann–Whitney *U* tests, or χ^2^ tests were used to compare demographic and clinical characteristics, with results reported as mean ± standard deviation or median (interquartile range), as appropriate. To control for potential confounders, differences in oculomotor phenotypes and ^18^F-FDG PET SUVRs were assessed using generalized linear models (GLMs) with adjustment for age and sex, selecting Gaussian or Gamma distributions according to data characteristics. Threshold determination was based on model residuals, with the residual cutoff corresponding to the maximum Youden index defined as the optimal diagnostic threshold; within the PD cohort, individuals were stratified by metric direction, with residuals ≥ cutoff classified as “abnormally increased” and residuals ≤ cutoff classified as “abnormally decreased.” Associations among clinical variables, oculomotor parameters, and brain metabolic features were evaluated using Pearson or Spearman correlation analyses. All statistical analyses were performed in a Python environment.

## Results

### Baseline characteristics

Baseline demographic and clinical characteristics of patients with PD (*n* = 122) and HCs (*n* = 69) are summarized in Table 1. The PD group was older than the HC group (65.00 [59.00–72.00] vs. 55.00 [40.00–62.00] years, *p* < 0.001), whereas sex distribution and years of education did not differ. PD patients had a median H&Y stage of 2.00 and a median disease duration of 4.50 years. Overall symptom measures was greater in the PD group, with higher motor and non-motor symptom severity and poorer quality of life (*p* < 0.01). The oculomotor–derived cognitive/motor score was significantly lower in PD than in HC participants (74.00 [66.00–82.75] vs. 81.00 [75.00–87.00], *p* < 0.001).

**TABLE 1:** Demographic and clinical characteristics of participants with Parkinson’s disease and healthy controls.

Variables	PD (*n* = 122)	HC (*n* = 69)	*P*-value
Age (years)	65.00 [59.00, 72.00]	55.00 [40.00, 62.00]	< 0.001
SEX (Male)	57	35	0.595
BMI	23.90 ± 3.21	24.50 ± 3.87	0.280
Weight	63.07 ± 10.56	64.75 ± 12.64	0.349
Height	160.00 [156.00, 169.75]	161.00 [155.00, 169.00]	0.960
**Education**			
Illiteracy (no schooling)	4	0	0.358
Primary school	15	11	
Junior high school	36	17	
Senior high school or above	67	41	
Hoehn & Yahr	2.00 [2.00, 2.50]	0.00 [0.00, 0.00]	< 0.001
LEDD	445.50 [300.00, 699.50]	0.00 [0.00, 0.00]	< 0.001
Disease year	4.50 [2.00, 6.75]	0.00 [0.00, 0.00]	< 0.001
PDQ-39	11.50 [6.00, 18.00]	4.00 [2.00, 6.00]	< 0.001
MDS-UPDRS-Part I	8.00 [5.00, 12.00]	3.00 [2.00, 7.00]	< 0.001
MDS-UPDRS-Part II	5.00 [3.00, 8.00]	2.00 [1.00, 3.00]	< 0.001
MDS-UPDRS-Part III	35.00 [27.00, 45.00]	0.00 [0.00, 2.00]	< 0.001
MDS-UPDRS-Part IV	0.00 [0.00, 2.00]	0.00 [0.00, 0.00]	< 0.001
MDS-UPDRS-Total (4 sections)	52.00 [41.00, 66.00]	7.00 [4.00, 14.00]	< 0.001
STAI	43.00 [42.00, 45.00]	41.00 [40.00, 43.00]	< 0.001
FSS	6.00 [4.00, 10.00]	4.00 [3.00, 6.00]	< 0.001
HAMA	8.00 [6.00, 13.00]	7.00 [5.00, 8.00]	< 0.001
GDS	7.00 [6.00, 8.75]	6.50 [5.00, 8.00]	0.004
HAMD	8.00 [6.00, 12.00]	7.00 [5.00, 9.00]	0.007
ESS	4.00 [3.00, 6.75]	4.00 [2.00, 6.00]	0.072
Scapa-aut	4.00 [2.00, 6.00]	4.00 [2.00, 6.00]	0.371
Cognitive scores	74.00 [66.00, 82.75]	81.00 [75.00, 87.00]	< 0.001
Movement scores	72.00 [62.00, 78.00]	79.00 [73.00, 82.00]	< 0.001

BMI, body mass index; H&Y, Hoehn & Yahr stage; LEDD, levodopa equivalent daily dose; PDQ-39, 39-item Parkinson’s Disease Questionnaire; MDS-UPDRS, Movement Disorder Society–Unified Parkinson’s Disease Rating Scale; STAI, State–Trait Anxiety Inventory; FSS, Fatigue Severity Scale; HAMA, Hamilton Anxiety Rating Scale; HAMD, Hamilton Depression Rating Scale; GDS, Geriatric Depression Scale; ESS, Epworth Sleepiness Scale; SCOPA-AUT, Scales for Outcomes in Parkinson’s Disease–Autonomic.

### Eye-movement differences between PD and HCs

**FIGURE 2:**
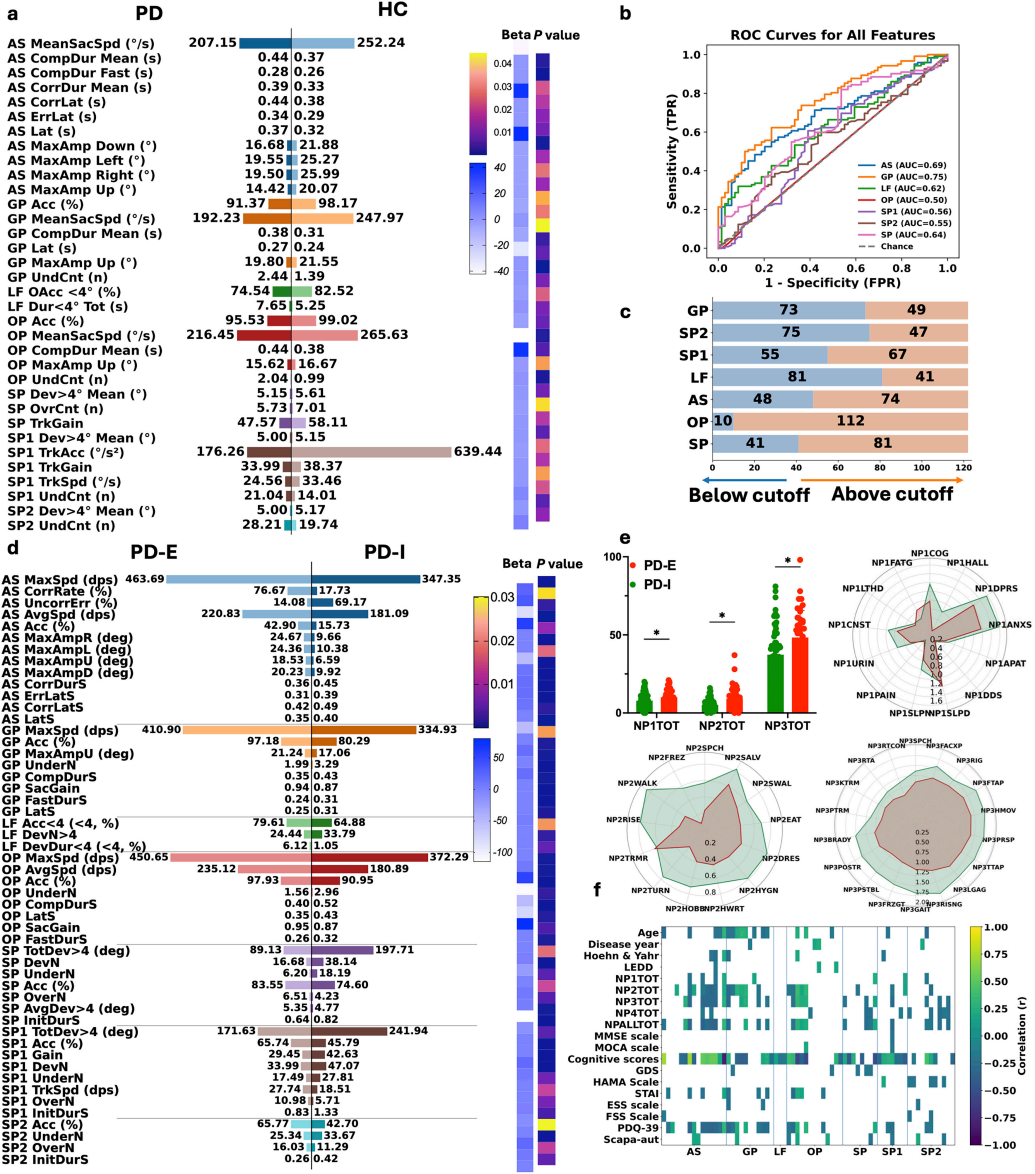
Multitask oculomotor–based group comparisons and subtype characterization in PD. **(a)** Comparison of multiple oculomotor parameters between patients with PD and HCs. Representative metrics derived from antisaccade (AS), smooth pursuit (GP, LF), overlap saccade (OP), and saccade/pursuit variant tasks (SP, SP1, SP2) are shown as group means. Color bars on the right indicate the magnitude of the regression coefficients (β) and the corresponding *P*-values, reflecting the strength and statistical significance of associations between oculomotor parameters and disease status. **(b)** Receiver operating characteristic (ROC) curve analyses based on features derived from individual oculomotor tasks. **(c)** Distribution of PD patients classified below and above the residual-based cutoff for each oculomotor task, illustrating subgroup allocation according to abnormality thresholds. **(d)** Comparison of oculomotor parameters between the two oculomotor subtypes, PD-E (efficient oculomotor control) and PD-I (inefficient oculomotor control). **(e)** Bar plots show group differences in MDS-UPDRS composite scores for NP1 (Part I: non-motor experiences of daily living), NP2 (Part II: motor experiences of daily living), and NP3 (Part III: motor examination). Radar plots further illustrate contrasts across individual subdomains/items within these components. **(f)** Heatmap showing correlations between oculomotor task–derived metrics and clinical characteristics.

Compared with HCs, the PD group showed the most pronounced impairments in the AS task, characterized by reduced prosaccadic velocity and amplitude and prolonged response times. In the GP task, PD patients exhibited lower accuracy and an increased number of undershoots, while overall accuracy was also reduced during LF tasks. During the OP task, PD patients demonstrated longer completion durations, lower mean saccadic velocity, reduced accuracy, and a higher frequency of undershoots. With respect to SP (SP), PD patients showed reduced tracking gain in both horizontal and vertical directions, accompanied by fewer overshoots and a corresponding increase in undershoots (Figure 2a).

### ROC analysis and diagnostic thresholds

After age and sex adjustment, one representative metric with the highest discriminative performance was selected from each eye-movement task for ROC analysis and threshold-based stratification (7 metrics in total). These metrics differentiated PD from HCs with AUC values ranging from 0.50 to 0.75. The GP-derived metric showed the highest discriminative performance, whereas overshoot count in the OP task showed the lowest performance and marked sample imbalance (Figures 2b,c).

Using residual thresholds, PD patients were further stratified for within-group analyses. Stratification based on the SP–derived metric showed that the high blink-rate group was older, had higher anxiety scores, and exhibited reduced lateral deviation load but lower pursuit gain. Stratification by AS completion time identified a prolonged-completion group characterized by generalized response slowing, reduced saccadic velocity, higher AS accuracy, and longer error latencies. Stratification by total deviation time in the LF task revealed that the high-deviation group showed more frequent and larger gaze deviations, reduced accuracy, and delayed pursuit initiation. Subtask-specific stratification further revealed that higher blink rates in 15° horizontal SP subtask (SP1) were associated with older age, female predominance, and increased pursuit acceleration, whereas higher blink rates in vertical SP subtask (SP2) were associated with lower daytime sleepiness, greater autonomic burden, reduced deviation load, and lower pursuit gain. Finally, stratification by GP completion time identified a subgroup with greater cognitive and functional burden [higher 39-item Parkinson’s Disease Questionnaire (PDQ-39) scores and MDS-UPDRS Parts I/II scores] and widespread oculomotor inefficiency, including prolonged latencies and completion times, reduced velocity and pursuit gain, and decreased overall accuracy (Supplementary Table 1).

### Data-driven oculomotor clusters in PD

In PD patients, 82 oculomotor features were screened for redundancy by correlation pruning (| *r*| > 0.80), yielding 67 representative features for consensus clustering. Across *k* = 2–5, a two-cluster solution (*k* = 2) provided the best overall performance in terms of stability gain, within-cluster consensus, and between-cluster separation (mean silhouette = 0.29), and showed a clear tendency toward separation on the principal component analysis (PCA) plane (Supplementary Figure 1). To ensure robustness against initialization bias, the k-means algorithm was run multiple times with different random initial centroids, and the solution with the lowest within-cluster sum of squares was retained. The clustering structure remained stable across repeated initializations.

### Clinical comparison of oculomotor cluster subtypes

Based on consensus clustering, two oculomotor phenotypes were identified among PD patients: PD-I (*n* = 42) and PD-E (*n* = 80)-with PD-I being older than PD-E (69.71 ± 8.82 vs. 62.84 ± 9.44 years; *p* = 0.00013), while disease duration and years of education were comparable between groups.

With respect to disease burden, PD-I exhibited significantly higher MDS-UPDRS Part I–III scores and total scores (Part I *p* = 0.007; Part II *p* = 0.001; Part III *p* = 1.25 × 10^−4^; total *p* = 1.72 × 10^−5^), as well as a modestly higher H&Y stage (*p* = 0.031). No significant differences were observed for MDS-UPDRS Part IV or LEDD. Cognitively, MMSE and MoCA scores did not differ significantly between subtypes; however, the eye-movement–derived cognitive score (EMDCS) was markedly higher in PD-E than in PD-I (79.50 vs. 64.00, *p* = 3.00 × 10^–12^). Most mood, sleep, and autonomic measures were comparable, although PD-I showed higher State–Trait Anxiety Inventory (STAI) scores (*p* = 0.0023) and worse quality of life, as reflected by higher 39-item PDQ-39 total scores (17.50 vs. 8.00, *p* = 1.30 × 10^−5^), indicating greater impairment (Table 2). Detailed comparisons of MDS-UPDRS Parts I–III are shown in Figure 2e and Supplementary Table 2.

**TABLE 2:** Clinical characteristics across oculomotor subtypes.

Variables		PD-I (*n* = 42)	PD-E (*n* = 80)	*P-*value
Demographics	Sex (male)	23 (54.8%)	34 (42.5%)	0.272
	Age (years)	69.71 ± 8.82	62.84 ± 9.44	< 0.001
	Disease year	5.00 (2.25, 8.00)	4.46 (2.00, 6.00)	0.379
	BMI	24.64 ± 3.26	23.52 ± 3.14	0.071
Education				0.108
	Illiteracy (no schooling)	2 (4.8%)	2 (2.5%)	
	Primary school	8 (19.0%)	7 (8.8%)	
	Junior high school	15 (35.7%)	21 (26.2%)	
	Senior high school or above	17 (40.5%)	50 (62.5%)	
MDS-UPDRS	MDS-UPDRS-Part I	10.00 (7.25, 12.00)	7.00 (4.75, 11.00)	0.007
	MDS-UPDRS-Part II	8.00 (4.00, 12.75)	4.00 (2.00, 7.00)	0.001
	MDS-UPDRS-Part III	47.50 (38.75, 58.75)	33.50 (26.75, 43.00)	0.000
	MDS-UPDRS-Part IV	0.00 (0.00, 4.75)	0.00 (0.00, 0.00)	0.303
	MDS-UPDRS-Total	69.00 (53.00, 92.50)	46.00 (38.00, 64.50)	0.000
	Hoehn & Yahr	2.00 (2.00, 3.00)	2.00 (2.00, 2.50)	0.031
	LEDD	475.00 (359.38, 684.62)	387.50 (212.50, 700.00)	0.229
MMSE scale		16.00 (14.25, 19.00)	16.00 (13.00, 19.00)	0.402
	ORI	5.00 (5.00, 6.00)	5.00 (4.00, 6.00)	0.312
	MEM	4.00 (3.00, 4.00)	4.00 (3.00, 4.00)	0.320
	ATT	3.00 (2.00, 3.00)	3.00 (2.00, 3.00)	0.477
	LAN	4.00 (3.00, 6.00)	4.00 (3.00, 5.00)	0.813
	VIS	0.00 (0.00, 0.00)	0.00 (0.00, 0.00)	0.336
MOCA scale		12.50 (10.25, 15.75)	11.00 (9.00, 16.00)	0.215
	VSE	2.00 (1.00, 2.00)	1.00 (1.00, 2.00)	0.737
	NAM	2.00 (1.00, 2.00)	2.00 (1.00, 2.00)	0.145
	ATT.1	2.00 (1.00, 2.75)	2.00 (1.00, 2.25)	0.787
	LAN.1	3.00 (2.25, 4.00)	3.00 (2.00, 4.00)	0.412
	ABS	1.00 (0.00, 1.00)	0.50 (0.00, 1.00)	0.191
	REC	1.00 (0.00, 1.00)	1.00 (0.00, 1.00)	0.927
	ORI.1	3.00 (3.00, 4.00)	3.00 (3.00, 4.00)	0.569
**Non-motor assessment**				
	ESS	5.50 (2.25, 9.50)	4.00 (3.00, 6.00)	0.134
	FSS	8.00 (4.00, 10.00)	6.00 (4.00, 10.00)	0.282
	HAMA	8.00 (7.00, 12.75)	8.00 (6.00, 13.25)	0.322
	HAMD	8.00 (7.00, 12.00)	7.00 (6.00, 10.50)	0.184
	GDS	7.00 (7.00, 8.75)	7.00 (6.00, 8.25)	0.299
	STAI	45.00 (43.00, 48.00)	43.00 (41.00, 45.00)	0.002
	Scapa-aut	5.50 (3.25, 7.50)	4.00 (2.00, 6.00)	0.061
	PDQ-39	17.50 (11.00, 31.00)	8.00 (5.75, 13.50)	< 0.001
**Derived oculomotor score**				
Lifestyle	Cognitive scores	64.00 (56.50, 68.00)	79.50 (72.00, 86.00)	< 0.001
	Movement scores	60.00 (53.00, 66.75)	76.50 (69.00, 79.25)	< 0.001
	Alcohol drinking	3 (7.1%)	3 (3.8%)	0.702
	Tea consumption	8 (19.0%)	20 (25.0%)	0.606
	Physical activity	13 (31.0%)	31 (38.8%)	0.513

MMSE, domain abbreviations; ORI, orientation; MEM, registration/recall (memory); ATT, attention and calculation; LAN, language; VIS, visuospatial/constructive ability. MoCA, domain abbreviations; VSE, visuospatial/executive; NAM, naming; ATT.1, attention; LAN.1, language; ABS, abstraction; REC, delayed recall; ORI.1, orientation. BMI, body mass index; MDS-UPDRS, Movement Disorder Society–Unified Parkinson’s Disease Rating Scale; LEDD, levodopa equivalent daily dose; ESS, Epworth Sleepiness Scale; FSS, Fatigue Severity Scale; HAMA, Hamilton Anxiety Rating Scale; HAMD, Hamilton Depression Rating Scale; GDS, Geriatric Depression Scale; STAI, State–Trait Anxiety Inventory; PDQ-39, Parkinson’s Disease Questionnaire-39.

### Oculomotor subtype differences and their clinical correlates

After age and sex adjustment and multiple-comparison correction, the subtypes diverged: PD-E showed preserved oculomotor control, whereas PD-I showed broader oculomotor impairment.

In the AS task, PD-E showed stronger inhibitory control and error correction than PD-I. PD-E had a higher correction rate (76.67% vs. 17.73%, *q* = 2.25 × 10^–28^), a lower uncorrected error rate (14.08% vs. 69.17%, *q* = 1.81 × 10^–21^), and higher accuracy (42.90% vs. 15.73%, *q* = 2.99 × 10^−5^). PD-E also showed larger amplitudes, faster saccades, and shorter error-response and correction times. In the OP task, PD-E showed higher accuracy (97.93% vs. 90.95%, *q* = 5.47 × 10^−4^), shorter latency (347.00 vs. 432.45 ms, *q* = 5.32 × 10^−6^), and shorter completion time (402.38 vs. 522.46 ms, *q* = 1.40 × 10^−8^). PD-E also had gain closer to ideal (0.95 vs. 0.87, *q* = 6.36 × 10^–3^) and less hypometria. In the GP task, PD-E showed higher accuracy (97.18% vs. 80.29%, *q* = 8.37 × 10^−5^), shorter latency and completion time, higher gain (0.94 vs. 0.87, *q* = 3.21 × 10^–3^), and fewer hypometric saccades (1.99 vs. 3.29, *q* = 8.43 × 10^–3^). During SP, PD-E had higher horizontal and vertical tracking accuracy (SP1: 65.74% vs. 45.79%, *q* = 2.09 × 10^−4^; SP2: 65.77% vs. 42.70%, *q* = 7.79 × 10^−8^), improved deviation metrics, and shorter initiation time (830.64 vs. 1331.25 ms, *q* = 1.01 × 10^−4^). PD-E showed fewer hypometric events but slightly more overshoots (*q* < 0.03). In the LF task, PD-E showed shorter deviation time within < 4° (6124.65 vs. 10548.39 ms, *q* = 1.03 × 10^–3^), higher accuracy (79.61% vs. 64.88%, *q* = 1.24 × 10^–3^), and fewer deviation events (24.44 vs. 33.79, *q* = 3.71 × 10^–2^) (Figure 2d).

Within the PD cohort, oculomotor metrics correlated with clinical features. Cognitive measures showed the strongest and most consistent associations: higher MoCA/MMSE total and domain scores (language, visuospatial, memory, attention, abstraction, orientation, naming) aligned with better AS/GP/OP performance. Higher cognition was linked to higher accuracy, shorter latency, and better completion. Higher MDS-UPDRS scores were linked to slower responses, longer completion, lower gain, and higher error rates, and PDQ-39 showed the same direction. Age showed moderate negative associations (e.g., lower AS accuracy and higher errors), whereas mood, sleep, and autonomic scales showed weaker correlations (Figure 2f).

### Subtype-specific FDG-PET metabolic differences

**FIGURE 3:**
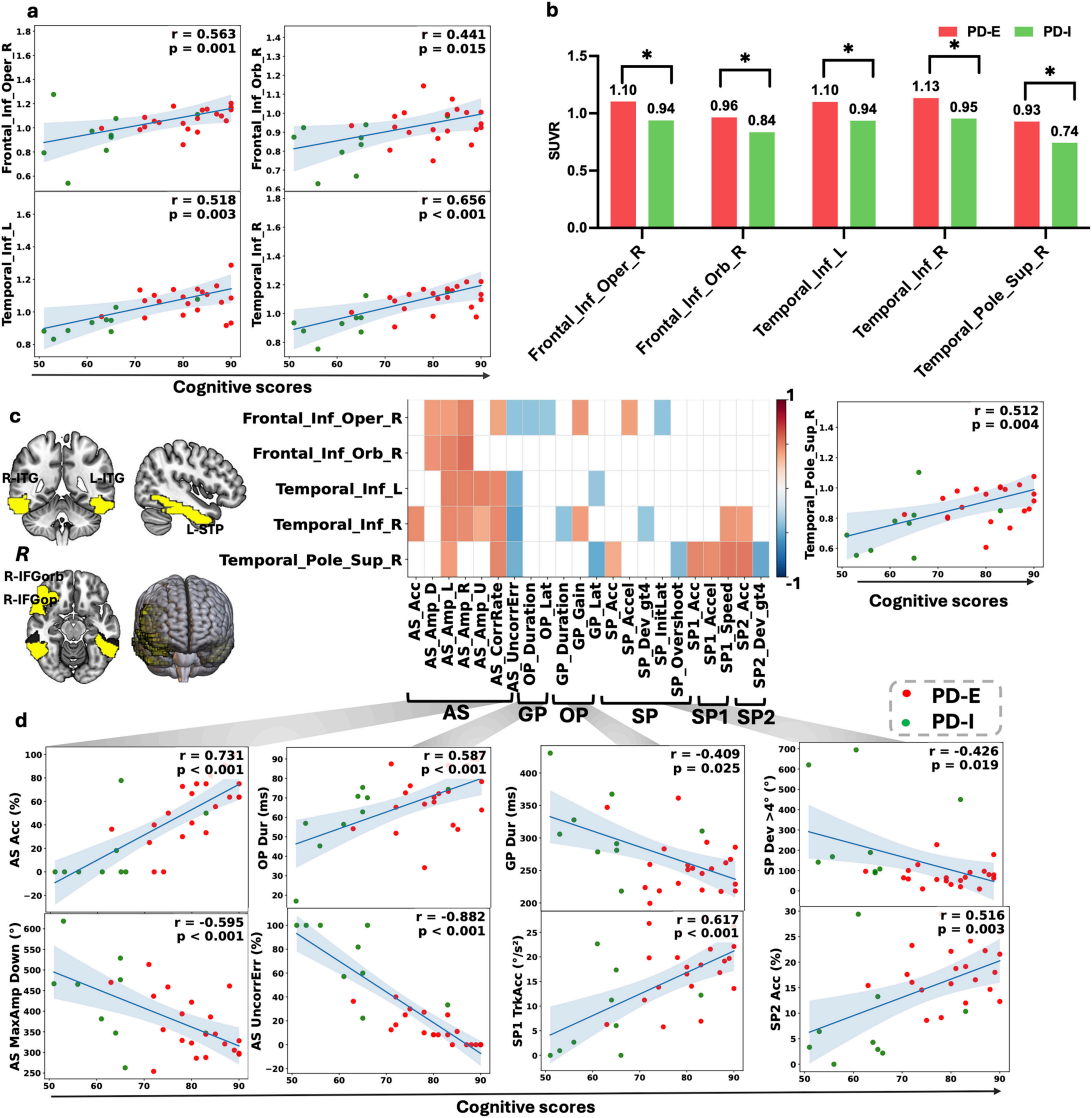
Associations between oculomotor performance, cerebral glucose metabolism, and cognitive function across PD subtypes. **(a)** Scatter plots illustrating correlations between regional ^18^F-FDG PET SUVR values and eye-movement–derived cognitive score (EMDCS). **(b)** Group comparisons of regional cerebral glucose metabolism between the two oculomotor subtypes. Bar plots display mean SUVR values (± SEM) for PD-E (red) and PD-I (green) in frontal and temporal regions, including the temporal pole. **(c)** Brain maps highlighting regions showing significant metabolic differences and/or associations with oculomotor and cognitive measures, alongside a heatmap summarizing correlations between selected regional SUVRs and representative oculomotor features derived from AS, GP, OP, and SP, SP1, SP2 tasks. Color intensity indicates the direction and magnitude of correlation coefficients. L-ITG, Inferior Temporal Gyrus, R-ITG, Inferior Temporal Gyrus, R-IFGop, Inferior Frontal Gyrus, opercular part, R-STP, Superior Temporal Pole, R-IFGorb, Inferior Frontal Gyrus, orbital part. **(d)** Scatter plots depicting relationships between key oculomotor parameters and EMDCS across tasks. Positive and negative correlations are shown for selected AS, GP, and SP/SP1/SP2 features (e.g., accuracy, duration, gain, and amplitude metrics). Each point represents an individual participant, colored by subtype.

Among the 30 PD patients with ^18^F-FDG PET, 9 were classified as PD-I and 21 as PD-E. Age- and sex-adjusted models showed that, compared with PD-I, PD-E exhibited relatively higher metabolic activity across several frontotemporal association cortices. Specifically, bilateral inferior temporal gyri showed significantly higher uptake in PD-E than in PD-I (Temporal_Inf_L: 1.099 vs. 0.936, β = 0.138, *p* = 0.0032; Temporal_Inf_R: 1.133 vs. 0.955, β = 0.149, *p* = 0.0034). The right inferior frontal operculum, right superior temporal pole, and right inferior frontal orbital gyrus also showed higher metabolism in PD-E (Frontal_Inf_Oper_R: 1.103 vs. 0.938, β = 0.144, *p* = 0.0345; Temporal_Pole_Sup_R: 0.928 vs. 0.743, β = 0.134, *p* = 0.0407; Frontal_Inf_Orb_R: 0.965 vs. 0.835, β = 0.104, *p* = 0.0418). However, none of these ROI differences survived multiple-comparison correction. PD-E also showed a non-significant trend toward lower cerebellar hemispheric uptake than PD-I (Figures 3b,c).

### FDG–cognitive–eye triad network

Regional glucose metabolism in the right inferior frontal operculum, bilateral inferior temporal gyri, and right temporal pole forms a stable “metabolism–cognition–eye movement” triad with EMDCS and key AS, OP, and SP/SP1 metrics.

In the right inferior frontal operculum, brain metabolism was moderately positively associated with AS correction rate, whereas EMDCS showed a stronger association with correction rate. Metabolism also correlated positively with maximal AS amplitudes across directions; In the inferior temporal gyri, correlations were broad and consistent: Left inferior temporal metabolism was moderately positively associated with AS correction rate and maximal amplitudes, and negatively associated with uncorrected error rate. EMDCS were strongly negatively associated with uncorrected errors. Inferior temporal metabolism was also negatively associated with the frequency and total magnitude of large horizontal SP deviations, as well as with OP initiation latency and mean completion duration; In the right temporal pole, higher metabolism was associated with larger leftward AS amplitude and lower uncorrected AS errors and OP initiation latency (Figure 3).

Cognitive subdomain analyses suggested that the triad was primarily driven by executive function, attention, and memory. The strongest contributions involved inhibition/planning, attentional allocation and switching, and short-term memory. Additional associations were observed with visuospatial and reaction abilities (Supplementary Table 3).

### Predictive modeling and Streamlit deployment for oculomotor subtype classification

Based on the prior clustering of PD subtypes using 67 oculomotor features, six key variables showing the strongest inter-cluster differences were selected from the AS task: correction rate, uncorrected error rate, and maximal saccade amplitudes in the up, down, left, and right directions. After feature standardization, model performance metrics—including AUC, accuracy, and F1 score from cross-validation and an independent test set—were summarized in Supplementary Table 4. All models achieved test-set AUC values > 0.8. Among them, the radial basis function SVM demonstrated the best generalization performance. Although L1-regularized logistic regression yielded a slightly higher AUC during cross-validation, its AUC and accuracy on the independent test set were inferior to those of the SVM model. Accordingly, SVM was selected as the final classifier for subsequent development and validation.

An interactive oculomotor subtype prediction platform was implemented using Streamlit. The web-based tool enables real-time prediction, single-case input, probabilistic visualization, and batch CSV upload with result export. A demonstration video of the complete workflow is provided (Supplementary Video 3).

## Discussion

This study used data-driven stratification of multi-task, high-dimensional oculomotor features to identify two PD subtypes. After age adjustment, subtype differences persisted in oculomotor speed/timing, deviation load, and AS error correction. PD-I showed a higher clinical burden, whereas PD-E was relatively milder. ^18^F-FDG PET further revealed frontotemporal metabolic differences, which, together with cognitive and eye-movement measures, formed a “metabolism–cognition– oculomotor” triad. These findings support oculomotor subtypes as quantifiable behavioral stratification markers and suggest links to cortical metabolic heterogeneity.

Previous studies indicate that oculomotor parameters relate to PD phenotypes and support early identification and subtype differentiation. However, few data-driven studies have derived subtypes from oculomotor features (Waldthaler et al., 2023), and their neurobiological basis remains insufficiently supported by multimodal evidence. This study compares clinical profiles across oculomotor subtypes and integrates ^18^F-FDG PET to link cross-regional metabolic differences with cognitive and oculomotor measures.

With respect to AS, early PD is consistently characterized by elevated error rates in complex oculomotor paradigms, which correlate with executive control; longitudinal studies further suggest that AS latency and error rates predict cognitive progression. The present study extends these findings by demonstrating that AS measures show the strongest associations with ^18^F-FDG metabolism and cognitive performance, and by refining the correspondence between specific oculomotor task features and clinical cognitive scales, thereby supporting AS as a sensitive behavioral phenotype of PD-related cognitive change (Liao et al., 2024; Voss et al., 2025). Beyond AS, specific saccadic paradigms (e.g., visually guided saccades) have also been linked to cognitive processes such as spatial working memory (ŞtefŞnescu et al., 2024), but also with affective symptoms and sleep-related disturbances (Popovic et al., 2025). Consistent with this, the current results indicate task-specific cognitive associations: GP showed greater discriminatory power between PD and HCs, whereas AS were more closely related to motor and non-motor burden. Although prior studies have reported reduced accuracy in memory-guided saccades (MGS) among patients with early PD, and have suggested that MGS serve as sensitive probes of spatial working memory and frontal executive function, MGS were not included in the present testing battery. Instead, we employed the AS paradigm as a cognitively demanding oculomotor task to assess inhibitory control, planning, and error monitoring—core executive domains that are frequently compromised in PD. Consistent with previous literature demonstrating impairment of visually guided saccades in PD, the present study extends this framework by decomposing visually guided paradigms into horizontal smooth pursuit, gap, and overlap conditions. This task-level refinement enhances resolution and allows more precise characterization of distinct components of visuomotor and executive dysfunction in PD (Popovic et al., 2026; Terao et al., 2011). In addition, previous evidence suggests that vertical saccadic abnormalities have higher specificity in differential diagnosis (Waldthaler et al., 2019). Moreover, studies integrating oculomotor features with AI approaches indicate potential gains in early cognitive impairment detection and risk stratification (Brien et al., 2023; Diotaiuti et al., 2025; Koch et al., 2024; Oyama et al., 2019). In line with these observations, the present study found associations between disease duration, disease severity, multidimensional cognitive measures, and multiple oculomotor features, suggesting that oculomotor phenotypes may reflect continuous variation in clinical burden and cognitive profiles in PD; however, their predictive validity requires further confirmation in standardized staging and longitudinal follow-up studies.

Previous studies suggest that impaired prosaccadic and AS performance may reflect deficits in frontal inhibitory control and dysfunction of related neural circuits (Halliday and Carpenter, 2010). Consistent with this, the PD-I subtype—characterized by a greater cognitive burden—showed reduced ^18^F-FDG uptake across multiple frontotemporal cortical regions. The distribution of these abnormalities matches known PD cognitive metabolic patterns, suggesting cortical metabolic involvement in this oculomotor subtype. In addition, individuals with greater cognitive burden exhibited a trend toward relatively increased cerebellar metabolism. In light of prior reports linking elevated cerebellar metabolism to poorer cognitive performance (Ren et al., 2025; Walker et al., 2018), this observation may reflect cerebellar metabolic alterations related to compensatory or network-level processes associated with cognitive impairment.

It should be noted that patients were not stratified according to established clinical cognitive stages, precluding an assessment of the independent utility of oculomotor measures for early cognitive impairment detection. Previous FDG-PET studies have also shown that hypometabolism in posterior cortical regions can precede the onset of cognitive symptoms and predict subsequent cognitive decline (Walker et al., 2018), and that in cognitively impaired PD patients, hypometabolism in fronto–parietal–cingulate (and posterior temporal) cortices parallels cognitive deterioration and may exhibit stage-related distribution patterns (Khomenko et al., 2020; Li M. et al., 2025; Chong et al., 2025).

More broadly, the substantial heterogeneity exists across studies in oculomotor task paradigms and in the operational definitions of derived metrics. Consequently, a single task or feature rarely achieves stable sensitivity, predictive validity, and reproducibility simultaneously, and it is unlikely to support a unified, standardized interpretive framework in isolation (Antoniades and Spering, 2024). Our findings corroborate this limitation: when the number of overshoots in the OP task was used as the sole stratification feature, the resulting groups were markedly imbalanced in sample size, indicating that threshold-based stratification is vulnerable to distributional skewness and lacks robustness.

In parallel, prior data-driven subtyping work has highlighted a non-monotonic phenotype correspondence between phenotypes, whereby oculomotor abnormalities do not necessarily map onto detectable differences in conventional cognitive scales (Waldthaler et al., 2023). Consistent with this, we observed that PD-E tended to be faster and more accurate across most tasks, yet exhibited opposite tendencies in certain SP corrective indices (e.g., slightly increased overshoots), underscoring that oculomotor phenotypes likely require a multi-task integrative evaluation and corroboration by multimodal evidence.

Importantly, no significant differences were observed between PD-E and PD-I in MMSE, MoCA total scores, or cognitive subdomains. With mean MMSE and MoCA scores of ∼16 and ∼11, the cohort overall suggests a moderate level of cognitive impairment. At this stage, subtypes derived from eye-movement features may be less able to further separate “global cognitive severity” as captured by brief screening instruments, implying limited clinical discriminability for stratifying moderate impairment. This pattern aligns directionally with Waldthaler et al. (2023), who similarly reported no clear cognitive separation across oculomotor subtypes; however, their cohort had a substantially higher MoCA mean (∼24), closer to mild or borderline impairment. In contrast, the lower cognitive performance in our sample increases the risk of floor effects and restricted range, further reducing the ability of MMSE/MoCA to detect between-subtype differences.

The present findings are consistent with the prevailing conceptual framework that cognitive impairment in PD predominantly affects executive function, attention, and working memory. According to the Movement Disorder Society (MDS) criteria for Parkinson’s disease mild cognitive impairment (PD-MCI), executive and attentional deficits are among the most frequently involved cognitive domains in the early and intermediate stages of PD, and often precede the emergence of overt dementia (Litvan et al., 2012). In our cohort, cognitive measures demonstrated the strongest and most consistent associations with oculomotor metrics, particularly within antisaccade and prosaccade paradigms. Importantly, subdomain analyses indicated that the metabolism–cognition–oculomotor triad was primarily driven by executive control, attentional allocation and shifting, and short-term memory processes. These components correspond to core functions of fronto–striatal circuits, including inhibitory control and planning, which are critically engaged during antisaccade execution and error correction.

Notably, although global screening instruments such as the MoCA and the MMSE did not differentiate PD-E and PD-I at the total-score level, domain-specific associations suggest that oculomotor phenotypes may capture continuous variation within executive–attentional networks that is not fully resolved by brief cognitive staging tools. This pattern aligns with the MDS Level I screening framework, which is optimized for detecting the presence of cognitive impairment but is less sensitive to subtle domain-level stratification. In contrast, task-based oculomotor measures inherently embed executive demands, thereby offering greater functional specificity. Additional associations with visuospatial and reaction abilities further support the view that PD-related cognitive dysfunction is multidimensional, yet hierarchically weighted toward executive–attentional processes. Collectively, these findings reinforce the interpretation that oculomotor phenotypes reflect the integrity of fronto–striatal executive networks and may serve as behavioral indicators of domain-specific cognitive variation across the PD cognitive spectrum, even in the absence of marked differences in global cognitive severity.

Nevertheless, the EMDCS constructed from features spanning SP, OP, and AS did differentiate PD-E from PD-I. Importantly, this score is not independent of the oculomotor information used for subtyping (Kriegeskorte et al., 2009); rather, it represents a classic case of data double use (“double dipping”) or a non-independent comparison, which can inflate apparent effect sizes and weaken its interpretability as evidence for an independent cognitive-phenotype distinction (Starcke et al., 2025).

Given the well-established effects of age on saccadic velocity, reaction time, and pursuit stability, age was treated as a necessary confounder in our analyses (Mack et al., 2020). Although age showed directionally consistent associations with multiple oculomotor indices, the key PD-E versus PD-I differences remained robust after adjustment and were concentrated in nodes plausibly related to executive control (e.g., error correction, deviation, and cross-task temporal efficiency). This pattern argues against a simple explanation based on age differences or non-specific generalized slowing. At the network level, PDRP is typically characterized by relatively increased metabolism in thalamus/brainstem/basal ganglia–motor cortex and cerebellum, with relative hypometabolism in parieto-occipital and frontal association regions; PDCP, in contrast, is marked by hypometabolism in fronto-parietal association cortices (e.g., dorsolateral prefrontal cortex, inferior parietal lobule, precuneus), often accompanied by relative hypermetabolism in portions of the cerebellum. Although nigrostriatal dopaminergic degeneration often exhibits lateralized asymmetry, PDRP/PDCP expression tends to be more bilaterally symmetric and increases with disease duration, suggesting that PD may reflect whole-brain metabolic network reconfiguration rather than purely lateralized pathway disruption (Tang et al., 2020).

Within this framework of large-scale metabolic network reorganization, the relatively higher glucose metabolism observed in the PD-E group compared with PD-I warrants a mechanistic interpretation. Importantly, such “relative hypermetabolism” should not be viewed in isolation, but rather in relation to the established topology of PDRP and PDCP. In early or less impaired stages of PD, increased metabolic activity in selected cortical association regions has been interpreted as compensatory recruitment, reflecting adaptive engagement of fronto-parietal executive networks in response to subcortical dopaminergic dysfunction (Sosnik et al., 2025). Functional imaging studies have shown that enhanced activity within dorsolateral prefrontal and parietal cortices may support task performance despite basal ganglia impairment, particularly in domains requiring cognitive control, response monitoring, and performance optimization (Yang et al., 2024).

Accordingly, the relatively higher metabolism in PD-E may signify preserved or more efficiently recruited executive–oculomotor circuitry. Given that the PD-E phenotype was characterized by superior cross-task temporal coordination and reduced error-related deviations, the metabolic pattern may reflect stronger cortico–striato–thalamo–cortical coupling that sustains performance efficiency. From a systems neuroscience perspective, this aligns with the concept that network-level resilience—rather than isolated regional integrity—underpins behavioral preservation in PD (Cascone et al., 2021). In this view, PD-E may represent a subtype with attenuated PDCP expression and relatively preserved fronto-parietal metabolic integrity, whereas PD-I may reflect a trajectory with earlier disruption of associative network metabolism.

An alternative, though not mutually exclusive, explanation is that the observed metabolic differences represent a distinct subtype-specific metabolic signature rather than purely compensatory activation. Prior work has demonstrated that expression strength of PDRP and PDCP varies across clinical phenotypes and correlates with symptom burden and progression (Rus et al., 2022). If PD-E exhibits comparatively lower pathological network expression (e.g., reduced PDCP load) alongside preserved cortical metabolism, this would further support the interpretation that the “efficient” phenotype is associated with a more adaptive network configuration rather than generalized hyperexcitability (Huang et al., 2007b).

Importantly, hypermetabolism in neurodegenerative disorders does not uniformly indicate favorable prognosis; in certain contexts it may reflect maladaptive overactivation or reduced neural efficiency (Camandola and Mattson, 2017). However, when considered alongside the behavioral profile and the localization of metabolic differences to executive-control–relevant regions, the present findings are more consistent with adaptive compensation or relative preservation of synaptic function.

The cognition–oculomotor–metabolism triad identified in the present study carries not only mechanistic significance but also provides a potentially actionable functional phenotyping framework. Notably, the correspondence between specific oculomotor paradigms and regional metabolic nodes demonstrates a relatively distinct network-level organization. AS metrics were primarily associated with temporal and parietal metabolic activity, suggesting that their functional basis extends beyond classical response inhibition to include goal representation updating, spatial integration, and sensory reorientation processes. In contrast, SP, OP, and SP1/SP2 metrics were more prominently linked to temporal lobe metabolic nodes, reflecting mechanisms related to visual target stabilization and sustained sensorimotor integration. GP metrics, however, showed stronger associations with frontal metabolic regions, indicating a greater dependence on prefrontal-mediated executive regulation and maintenance processes. This structured distribution supports conceptualizing the triad as multiple interrelated yet functionally differentiated regulatory systems, rather than as a unitary motor execution abnormality.

Furthermore, this structured framework provides a testable theoretical basis for mechanism-informed intervention matching. For instance, patients presenting predominantly with AS abnormalities—suggesting impairments in spatial goal updating or cognitive reorientation—may theoretically benefit from training programs targeting attentional shifting or spatial integration (Van Der Stigchel et al., 2011). Those exhibiting marked SP1/SP2 instability may be better suited for visuomotor predictive control training (Miyamoto et al., 2021). More pronounced GP abnormalities may indicate involvement of frontal executive maintenance networks and could therefore represent potential targets for executive function reinforcement or neuromodulation-based interventions (He et al., 2021). Although these implications require prospective validation, the metabolism–cognition–oculomotor coupling observed in this study offers a clearly defined hypothesis-generating framework for selecting intervention strategies based on network-specific functional characteristics.

In the machine learning analyses, the present study demonstrates that reliable prediction of vulnerable PD subtypes at the individual level can be achieved using only six parameters extracted from the AS task. This finding indicates that robust subtype discrimination may not require an extensive array of oculomotor paradigms; rather, a streamlined task design emphasizing cognitively demanding oculomotor control may be sufficient to capture core phenotypic differences in eye-movement efficiency, clinical burden, and cerebral metabolic status among patients with PD. From a translational medicine perspective, achieving stable subtype classification with a limited set of AS-derived features reduces reliance on the specialized neurological expertise typically required for conventional clinical rating scales. Consequently, this approach may facilitate large-scale, objective screening and stratification of PD patients, particularly in regions with limited access to trained movement-disorder specialists (Koerner et al., 2025). Collectively, these findings support the potential utility of AS-based eye-movement metrics as a scalable and clinically feasible tool for standardized phenotypic profiling in PD.

Several limitations warrant consideration. First, not all participants underwent ^18^F-FDG PET, constraining the sample size for multimodal analyses. Nonetheless, within the PET subsample, the oculomotor subtype differences observed in the full cohort were recapitulated, supporting internal consistency; however, missingness may still introduce selection bias and reduce statistical power, thereby affecting stability and generalizability. Second, the oculomotor battery was relatively simplified and administered under fixed, controlled experimental conditions; its correspondence to real-world oculomotor control in dynamic visual environments remains uncertain, potentially limiting ecological validity. Third, we lacked pathology-specific molecular imaging or fluid biomarkers (e.g., Aβ/τ PET or dopaminergic functional imaging), precluding inference about differential co-pathology burden or dopaminergic impairment across subtypes and limiting mechanistic attribution. Finally, the study is cross-sectional. Ongoing follow-up will leverage longitudinal data to evaluate the predictive validity of this data-driven subtyping and key oculomotor measures for cognitive and clinical progression, and to test reproducibility and translational value across disease stages and larger cohorts.

The findings of the present study open several promising avenues for advancing mechanism-based personalized therapeutic strategies in PD. First, future research should further refine a mechanistic framework centered on the triadic coupling of cognition, oculomotor function, and brain metabolism. Longitudinal follow-up and multimodal integrative analyses will be essential to delineate the causal relationships and dynamic evolution among distinct network nodes, thereby enabling a more precise characterization of functional reorganization trajectories in Parkinson’s disease. Such efforts may help distinguish stable phenotypic traits from stage-dependent alterations and provide a stronger biological foundation for mechanism-informed stratification. Second, the novel oculomotor subtypes identified in this study warrant independent validation in larger and multicenter cohorts. Future work should assess the reproducibility and stability of subgroups dominated by specific oculomotor dimensions and examine their associations with cognitive trajectories, motor progression, and other biological markers. This approach may facilitate the development of a circuit-informed subtyping framework that moves beyond traditional symptom-based classifications. Finally, building upon mechanistic refinement and subtype validation, clinical translation represents a critical next step. Oculomotor–metabolic indices could be incorporated as stratification variables or sensitive outcome measures in future clinical trials to determine whether distinct network profiles correspond to differential treatment responses. Through mechanism-driven stratification and intervention matching, it may become possible to establish more precise and empirically testable personalized therapeutic strategies for PD.

Overall, using data obtained from non-invasive eye-tracking, this study identified two PD subtypes with distinct phenotypic profiles. The corresponding subtype-specific alterations in brain metabolism further suggest an interpretable concordance between oculomotor phenotypes and large-scale metabolic brain networks. These findings support the potential utility of multi-task oculomotor measures as clinical biomarkers reflecting brain functional reorganization, with relevance for characterizing PD heterogeneity and for objective risk stratification and disease monitoring.

## Data Availability

The original contributions presented in this study are included in this article/Supplementary material, further inquiries can be directed to the corresponding authors.
