# Supplementary material for: Multimodal data-driven eye-movement subtypes and their cerebral glucose metabolic patterns in Parkinson’s disease
Source: Front Aging Neurosci. 2026 Mar 11;18:1794652. doi: 10.3389/fnagi.2026.1794652 (PMC13013447; doi:10.3389/fnagi.2026.1794652)
Supplement: Supplementary file 1 [file Supplementary_file_1.pdf]

Supplemental Table 1.1 Residual-based thresholds (Youden index) for defining below- and above-threshold groups across eye-movement tasks

Eye move task	Task domain	Grouping feature (residual)	Residual cutoff (Youden)	Below-threshold group	Above-threshold group
SP	Horizontal Smooth Pursuit (15°)	SP_EyeOpeningRate_pct	1.32	Residual < 1.32 (Low eye-opening rate)	Residual ≥ 1.32 (High eye-opening rate)
OP	Overlap Prosaccade	OP_OvershootCount_n	-0.18	Residual < -0.18 (Low overshoot)	Residual ≥ -0.18 (High overshoot)
AS	Antisaccade	AS_CompletionDurationAvg_ms	-18.7	Residual < -18.7 (Short completion)	Residual ≥ -18.7 (Long completion)
LF	Lateral Fixation	LF_TotalDeviationDuration_lt4deg_ms	1783.9	Residual < 1783.9 (Short deviation duration)	Residual ≥ 1783.9 (Long deviation duration)
SP1	Horizontal Smooth Pursuit (15°)	SP1_EyeOpeningRate_pct	1.6	Residual < 1.6 (Low eye-opening rate)	Residual ≥ 1.6 (High eye-opening rate)
SP2	Vertical Smooth Pursuit (15°)	SP2_EyeOpeningRate_pct	1.87	Residual < 1.87 (Low eye-opening rate)	Residual ≥ 1.87 (High eye-opening rate)
GP	Gap Prosaccade	GP_CompletionDurationAvg_ms	31.9	Residual < 31.9 (Short completion)	Residual ≥ 31.9 (Long completion)

Supplemental Table 1.2 Eye-movement features differing between below- and above-threshold residual groups across tasks

Eye movement tasks	feature	Below-threshold group	Above-threshold group	beta	se	*P* value	p_fdr
AS	AS_Accuracy_pct	15.55 ± 25.99	45.22 ± 27.62	27.23	4.92	<0.001	<0.001
AS	AS_AvgSaccadeSpeed_dps	249.67 ± 69.96	179.56 ± 69.13	-72.74	12.52	<0.001	<0.001
AS	AS_CompletionDurationAvg_ms	346.05 ± 42.29	501.08 ± 95.01	158.15	12.24	<0.001	<0.001
AS	AS_CompletionDurationFastest_ms	247.01 ± 33.48	307.10 ± 78.08	0.22	0.04	<0.001	<0.001
AS	AS_CorrectResponseLatency_ms	408.71 ± 67.30	470.42 ± 131.59	0.15	0.04	<0.001	<0.001
AS	AS_ErrorResponseLatency_ms	295.96 ± 51.12	368.17 ± 119.30	0.22	0.04	<0.001	<0.001
AS	AS_Latency_ms	306.32 ± 49.94	410.18 ± 91.14	105.15	12.59	<0.001	<0.001
AS	AS_MaxSaccadeSpeed_dps	482.77 ± 182.58	385.29 ± 155.89	-0.25	0.07	<0.001	<0.001
AS	LF_OverallAccuracy_lt4deg_pct	79.75 ± 15.12	71.16 ± 20.88	-9.08	3.22	<0.001	0.03
AS	GP_Latency_ms	250.47 ± 49.29	284.68 ± 63.18	0.14	0.04	<0.001	<0.001
AS	OP_CompletionDurationAvg_ms	402.12 ± 89.18	470.70 ± 100.85	75.61	16.49	<0.001	<0.001
AS	OP_CompletionDurationFastest_ms	252.33 ± 60.45	301.63 ± 69.73	51.99	11.29	<0.001	<0.001
AS	OP_Latency_ms	336.59 ± 68.34	402.26 ± 83.20	72.67	12.94	<0.001	<0.001
AS	OP_MaxSaccadeSpeed_dps	459.37 ± 124.27	400.51 ± 107.55	-63.05	22.11	<0.001	0.03
AS	SP1_TrackingAcceleration_dps2	38.16 ± 47.34	265.85 ± 1226.09	1.48	0.45	<0.001	0.01
GP	AS_CompletionDurationAvg_ms	414.23 ± 96.49	478.60 ± 116.45	61.64	19.17	<0.001	0.01
GP	AS_CorrectionRate_pct	62.74 ± 35.78	46.91 ± 39.01	-15.90	6.52	0.01	0.04
GP	AS_CorrectResponseLatency_ms	418.62 ± 87.92	487.14 ± 136.44	0.15	0.05	<0.001	0.01
GP	AS_ErrorResponseLatency_ms	316.82 ± 81.47	373.92 ± 124.17	0.16	0.05	<0.001	0.01
GP	AS_Latency_ms	345.43 ± 80.09	404.92 ± 99.17	57.45	16.55	<0.001	<0.001
GP	AS_MaxAmplitudeLeft_deg	21.72 ± 9.77	16.31 ± 9.64	-5.32	1.71	<0.001	0.01
GP	AS_MaxAmplitudeRight_deg	22.25 ± 10.11	15.41 ± 9.51	-6.73	1.70	<0.001	<0.001
GP	AS_MaxAmplitudeUp_deg	16.10 ± 9.61	11.92 ± 8.10	-4.21	1.52	0.01	0.02

GP	LF_OverallAccuracy_lt4deg_pct	78.70 ± 17.65	68.35 ± 19.97	-10.19	3.56	<0.001	0.02
GP	LF_TotalDeviationDuration_lt4deg_ms	6399.25 ± 5301.78	9507.34 ± 5995.26	0.41	0.13	<0.001	0.01
GP	GP_Accuracy_pct	94.39 ± 13.28	86.87 ± 19.51	-7.02	2.95	0.02	0.05
GP	GP_AvgSaccadeSpeed_dps	223.00 ± 64.24	146.40 ± 72.37	-75.99	12.03	<0.001	<0.001
GP	GP_CompletionDurationAvg_ms	325.35 ± 47.37	462.42 ± 91.64	0.35	0.03	<0.001	<0.001
GP	GP_CompletionDurationFastest_ms	237.11 ± 39.76	300.28 ± 109.92	0.23	0.05	<0.001	<0.001
GP	GP_Latency_ms	251.35 ± 49.18	300.83 ± 63.53	0.18	0.04	<0.001	<0.001
GP	GP_MaxAmplitudeUp_deg	20.71 ± 5.02	18.44 ± 4.82	-2.19	0.85	0.01	0.03
GP	GP_MaxSaccadeSpeed_dps	412.23 ± 135.40	343.80 ± 149.10	-0.18	0.07	0.01	0.02
GP	GP_SaccadeGain	0.94 ± 0.09	0.89 ± 0.10	-0.05	0.02	<0.001	0.01
GP	GP_UndershootCount_n	1.90 ± 2.06	3.24 ± 1.96	1.32	0.36	<0.001	<0.001
GP	OP_Accuracy_pct	98.24 ± 5.21	91.50 ± 11.21	-6.55	1.65	<0.001	<0.001
GP	OP_AvgSaccadeSpeed_dps	237.63 ± 67.59	184.90 ± 65.62	-52.19	12.19	<0.001	<0.001
GP	OP_CompletionDurationAvg_ms	404.89 ± 82.55	501.56 ± 100.91	95.70	16.00	<0.001	<0.001
GP	OP_CompletionDurationFastest_ms	267.28 ± 54.99	304.52 ± 84.06	36.53	13.09	0.01	0.02
GP	OP_Latency_ms	350.73 ± 67.60	414.69 ± 91.45	64.16	14.38	<0.001	<0.001
GP	OP_MaxSaccadeSpeed_dps	444.44 ± 115.28	392.72 ± 115.09	-52.42	21.32	0.01	0.04
GP	OP_SaccadeGain	0.94 ± 0.10	0.89 ± 0.10	-0.05	0.02	<0.001	0.01
GP	OP_UndershootCount_n	1.55 ± 1.83	2.78 ± 2.12	1.20	0.36	<0.001	0.01
GP	SP1_InitiationDuration_ms	858.10 ± 447.37	1218.83 ± 931.24	0.34	0.13	0.01	0.02
GP	SP1_OverallAccuracy_pct	63.81 ± 21.07	51.51 ± 23.48	-12.81	4.07	<0.001	0.01
GP	SP2_OverallAccuracy_pct	62.42 ± 19.57	50.98 ± 19.88	-11.87	3.54	<0.001	0.01
LF	LF_DeviationCount_gt4deg_n	22.69 ± 14.94	37.48 ± 22.73	0.51	0.12	<0.001	<0.001
LF	LF_OverallAccuracy_lt4deg_pct	85.18 ± 7.86	53.53 ± 17.67	-31.41	2.87	<0.001	<0.001
LF	LF_TotalDeviation_gt4deg_deg	178.88 ± 78.13	243.23 ± 131.31	0.31	0.10	<0.001	0.01
LF	LF_TotalDeviationDuration_lt4deg_ms	4452.85 ± 2362.18	13959.13 ± 5305.60	1.15	0.08	<0.001	<0.001

LF	SP_OverallAccuracy_pct	83.27 ± 16.24	74.94 ± 15.48	-8.63	2.91	<0.001	0.02
LF	SP1_AvgDeviation_gt4deg_deg	4.91 ± 0.43	5.18 ± 0.52	0.06	0.02	<0.001	0.02
LF	SP1_InitiationDuration_ms	842.55 ± 401.56	1319.93 ± 1009.31	0.44	0.13	<0.001	0.01
LF	SP1_OverallAccuracy_pct	66.52 ± 18.88	43.77 ± 22.50	-22.75	3.98	<0.001	<0.001
LF	SP2_AvgDeviation_gt4deg_deg	4.83 ± 0.44	5.34 ± 0.97	0.10	0.03	<0.001	<0.001
LF	SP2_InitiationDuration_ms	241.57 ± 258.25	463.67 ± 548.02	0.66	0.21	<0.001	0.02
LF	SP2_OverallAccuracy_pct	63.51 ± 18.46	46.59 ± 19.57	-16.75	3.55	<0.001	<0.001
LF	SP2_TrackingGain	30.61 ± 23.70	53.05 ± 39.89	0.58	0.14	<0.001	<0.001
SP	LF_DeviationCount_gt2deg_n	63.68 ± 36.39	42.67 ± 24.87	-0.43	0.11	<0.001	<0.001
SP	LF_DeviationCount_gt4deg_n	35.93 ± 22.37	23.48 ± 15.90	-0.50	0.12	<0.001	<0.001
SP	LF_SquareWaveJerksCount_n	31.44 ± 26.83	18.86 ± 15.49	-13.82	4.86	<0.001	0.03
SP	LF_TotalDeviation_gt4deg_deg	251.63 ± 120.52	174.63 ± 82.79	-0.42	0.09	<0.001	<0.001
SP	SP_EyeOpeningRate_pct	93.30 ± 10.50	98.13 ± 0.44	5.56	1.79	<0.001	0.02
SP	SP_TrackingGain	63.15 ± 36.34	39.69 ± 22.64	-0.50	0.11	<0.001	<0.001
SP	SP1_DeviationCount_n	47.02 ± 33.15	34.17 ± 17.70	-0.35	0.13	0.01	0.05
SP	SP1_TotalDeviation_gt4deg_deg	247.40 ± 193.72	169.73 ± 90.82	-0.42	0.14	<0.001	0.02
SP	SP1_TrackingGain	45.22 ± 35.65	28.30 ± 16.85	-0.56	0.13	<0.001	<0.001
SP	SP2_DeviationCount_n	54.29 ± 32.27	38.35 ± 14.65	-0.37	0.10	<0.001	<0.001
SP	SP2_TotalDeviation_gt4deg_deg	277.94 ± 183.87	192.99 ± 90.53	-0.40	0.11	<0.001	<0.001
SP	SP2_UndershootCount_n	35.49 ± 23.78	24.52 ± 11.64	-0.38	0.12	<0.001	0.01
SP1	SP1_EyeOpeningRate_pct	93.42 ± 11.01	98.10 ± 0.31	5.77	1.74	<0.001	0.02
SP1	SP1_TrackingAcceleration_dps2	33.84 ± 45.79	293.18 ± 1286.36	1.99	0.49	<0.001	<0.001
SP1	SP1_TrackingGain	43.39 ± 33.33	26.26 ± 13.75	-0.59	0.11	<0.001	<0.001
SP2	LF_DeviationCount_gt4deg_n	32.25 ± 21.88	20.34 ± 10.36	-0.49	0.11	<0.001	<0.001
SP2	LF_TotalDeviation_gt4deg_deg	226.43 ± 118.63	159.13 ± 50.76	-0.39	0.08	<0.001	<0.001
SP2	SP_DeviationCount_n	27.85 ± 26.89	18.03 ± 14.35	-12.84	4.48	<0.001	0.03
SP2	SP_EyeOpeningRate_pct	95.59 ± 8.13	97.97 ± 0.54	2.83	1.05	0.01	0.04

SP2	SP_TotalDeviation_gt4deg_deg	150.02 ± 148.73	89.00 ± 69.78	-79.32	24.31	<0.001	0.01
SP2	SP_TrackingGain	54.41 ± 32.28	36.65 ± 22.15	-0.44	0.11	<0.001	<0.001
SP2	SP1_DeviationCount_n	42.80 ± 29.23	31.62 ± 12.04	-0.31	0.10	<0.001	0.02
SP2	SP1_TotalDeviation_gt4deg_deg	220.88 ± 165.41	155.87 ± 62.26	-0.36	0.11	<0.001	0.01
SP2	SP1_TrackingGain	38.90 ± 29.62	26.15 ± 15.97	-0.46	0.12	<0.001	<0.001
SP2	SP2_AvgDeviation_gt4deg_deg	5.14 ± 0.81	4.78 ± 0.41	-0.09	0.02	<0.001	<0.001
SP2	SP2_DeviationCount_n	48.05 ± 26.81	36.77 ± 13.90	-0.28	0.09	<0.001	0.01
SP2	SP2_EyeOpeningRate_pct	94.55 ± 8.47	98.12 ± 0.64	3.98	1.06	<0.001	<0.001
SP2	SP2_TotalDeviation_gt4deg_deg	249.87 ± 156.30	176.33 ± 72.29	-0.37	0.10	<0.001	<0.001
SP2	SP2_TrackingGain	47.82 ± 35.38	22.72 ± 15.78	-0.81	0.13	<0.001	<0.001

Group differences were evaluated using regression models, with group (above-threshold vs below-threshold) entered as the predictor. Reported β coefficients represent the estimated mean difference (above-threshold minus below-threshold) for each feature, with corresponding standard errors (SE). Two-sided p values are shown, and p_fdr denotes false discovery rate–adjusted p values to account for multiple comparisons. Task abbreviations: AS, antisaccade; GP, gap prosaccade; OP, overlap prosaccade; LF, lateral fixation; SP, smooth pursuit; SP1/SP2, smooth pursuit subparadigms.

Supplemental Table 1.3 Clinical characteristics associated with residual-defined below- and above-threshold groups across eye-movement task domains

Task domain	Clinical feature	Below-threshold group	Above-threshold group	p_value
SP	Age (years)	61.44 ± 10.62	67.11 ± 8.77	0.004
SP	HAMA	12.00 (7.00–14.00)	8.00 (6.00–11.00)	0.032
SP	STAI	45.00 (42.00–46.00)	43.00 (41.00–45.00)	0.046
OP	Age (years)	53.80 ± 4.66	66.22 ± 9.46	<0.001
AS	Movement scores	76.00 (66.75–79.25)	68.50 (60.00–77.00)	0.009
LF	Movement scores	77.00 (69.00–80.00)	60.00 (53.00–67.00)	<0.001
SP1	Sex (male)	34 (61.8%)	23 (34.3%)	0.004
SP2	Age	63.20 ± 11.10	66.85 ± 8.24	0.046
SP2	ESS	5.00 (3.50–8.00)	4.00 (2.00–6.00)	0.020
SP2	Scapa-aut	4.00 (2.00–6.00)	5.00 (4.00–7.00)	0.020
SP2	Sex (male)	41 (54.7%)	16 (34.0%)	0.042
SP2	Age	63.80 ± 9.78	67.45 ± 9.42	0.043
GP	Movement scores	77.00 (68.00–81.00)	62.00 (56.00–70.00)	<0.001
GP	Cognitive scores	77.00 (68.00–85.00)	71.00 (63.00–78.00)	0.004
GP	PDQ-39	9.00 (6.00–16.00)	14.00 (8.00–23.00)	0.014
GP	MDS-UPDRS Part II score	4.00 (3.00–8.00)	7.00 (4.00–10.00)	0.046
GP	MDS-UPDRS Part I score	8.00 (4.00–11.00)	9.00 (6.00–12.00)	0.047

Supplemental Table 2. MDS-UPDRS Part I–III item- and total-score comparisons between oculomotor subtypes (PD-I vs PD-E)

Variable	PD-I (42)	PD-E (80)	*P*_value
**NP1**			
NP1COG	1.000 [1.000, 2.000]	1.000 [<0.001, 1.000]	0.009
NP1HALL	<0.001 [<0.001, 1.000]	<0.001 [<0.001, <0.001]	<0.001
NP1DPRS	1.500 [1.000, 2.000]	1.000 [<0.001, 2.000]	0.054
NP1ANXS	2.000 [1.000, 2.000]	1.000 [<0.001, 2.000]	0.035
NP1APAT	<0.001 [<0.001, 1.000]	<0.001 [<0.001, 1.000]	0.707
NP1DDS	<0.001 [<0.001, <0.001]	<0.001 [<0.001, <0.001]	0.913
NP1SLPD	1.000 [<0.001, 2.000]	1.000 [<0.001, 2.000]	0.633
NP1SLPN	<0.001 [<0.001, 2.000]	<0.001 [<0.001, 1.000]	0.380
NP1PAIN	<0.001 [<0.001, <0.001]	<0.001 [<0.001, <0.001]	0.965
NP1URIN	<0.001 [<0.001, 2.000]	<0.001 [<0.001, 1.000]	0.069
NP1CNST	0.500 [<0.001, 2.000]	<0.001 [<0.001, 2.000]	0.260
NP1LTHD	<0.001 [<0.001, 1.000]	<0.001 [<0.001, <0.001]	0.328
NP1FATG	<0.001 [<0.001, <0.001]	<0.001 [<0.001, 2.000]	0.139
NP1RTOT	9.500 [7.000, 12.000]	7.000 [5.000, 11.000]	0.017
**NP2**			
NP2SPCH	<0.001 [<0.001, 1.000]	<0.001 [<0.001, <0.001]	0.034
NP2SALV	<0.001 [<0.001, 2.000]	<0.001 [<0.001, 1.000]	0.300
NP2SWAL	<0.001 [<0.001, 1.000]	<0.001 [<0.001, 1.000]	0.310
NP2EAT	1.000 [<0.001, 1.000]	<0.001 [<0.001, 1.000]	0.064
NP2DRES	1.000 [<0.001, 1.000]	<0.001 [<0.001, 1.000]	0.003
NP2HYGN	1.000 [<0.001, 1.000]	<0.001 [<0.001, 1.000]	0.002
NP2HWRT	1.000 [<0.001, 1.000]	<0.001 [<0.001, 1.000]	0.051
NP2HOBB	1.000 [<0.001, 1.000]	<0.001 [<0.001, 1.000]	0.007
NP2TURN	1.000 [<0.001, 1.000]	<0.001 [<0.001, <0.001]	<0.001

NP2TRMR	<0.001 [<0.001, 1.000]	<0.001 [<0.001, 1.000]	0.318
NP2RISE	1.000 [<0.001, 1.000]	<0.001 [<0.001, 1.000]	0.003
NP2WALK	1.000 [<0.001, 1.000]	<0.001 [<0.001, 0.250]	<0.001
NP2FREZ	<0.001 [<0.001, 1.000]	<0.001 [<0.001, <0.001]	<0.001
NP2PTOT	8.000 [4.000, 12.750]	4.000 [2.750, 7.000]	0.002
**NP3**			
NP3SPCH	1.000 [1.000, 2.000]	1.000 [1.000, 1.000]	0.031
NP3FACXP	2.000 [1.000, 2.000]	1.000 [1.000, 2.000]	0.007
NP3RIGN	1.000 [1.000, 2.000]	1.000 [1.000, 2.000]	0.014
NP3RIGRU	1.000 [1.000, 2.000]	1.000 [1.000, 2.000]	0.057
NP3RIGLU	1.000 [1.000, 2.000]	1.000 [1.000, 2.000]	0.065
NP3RIGRL	1.000 [1.000, 2.000]	1.000 [1.000, 2.000]	0.093
NP3RIGLL	1.000 [1.000, 2.000]	1.000 [1.000, 2.000]	0.041
NP3FTAPR	2.000 [1.000, 2.000]	1.000 [1.000, 2.000]	0.003
NP3FTAPL	2.000 [1.000, 2.000]	1.000 [1.000, 2.000]	0.001
NP3HMOVR	2.000 [1.000, 2.000]	1.000 [1.000, 2.000]	0.002
NP3HMOVL	2.000 [1.000, 2.000]	1.000 [1.000, 2.000]	0.004
NP3PRSPR	2.000 [1.000, 2.000]	1.000 [1.000, 2.000]	0.007
NP3PRSPL	2.000 [1.000, 2.000]	1.000 [1.000, 2.000]	0.003
NP3TTAPR	2.000 [1.000, 2.000]	1.000 [1.000, 2.000]	0.003
NP3TTAPL	2.000 [1.000, 2.000]	1.000 [1.000, 2.000]	0.001
NP3LGAGR	2.000 [1.000, 2.000]	1.000 [1.000, 2.000]	0.002
NP3LGAGL	2.000 [1.000, 2.000]	1.000 [1.000, 2.000]	0.007
NP3RISNG	2.000 [1.000, 2.000]	1.000 [1.000, 1.250]	<0.001
NP3GAIT	2.000 [1.000, 2.000]	1.000 [1.000, 1.000]	<0.001
NP3FRZGT	2.000 [1.000, 2.000]	1.000 [1.000, 1.000]	<0.001
NP3PSTBL	2.000 [1.000, 2.000]	1.000 [1.000, 1.000]	<0.001
NP3POSTR	2.000 [1.000, 2.000]	1.000 [1.000, 1.000]	0.001

NP3BRADY	2.000 [1.000, 2.000]	1.000 [1.000, 1.000]	0.001
NP3PTRMR	1.000 [1.000, 2.000]	1.000 [<0.001, 1.000]	0.054
NP3PTRML	1.000 [<0.001, 2.000]	1.000 [<0.001, 1.000]	0.060
NP3KTRMR	1.000 [<0.001, 2.000]	1.000 [<0.001, 1.000]	0.089
NP3KTRML	1.000 [<0.001, 2.000]	1.000 [<0.001, 1.000]	0.266
NP3RTARU	1.000 [1.000, 2.000]	1.000 [<0.001, 1.000]	0.079
NP3RTALU	1.000 [<0.001, 2.000]	1.000 [<0.001, 1.000]	0.056
NP3RTARL	1.000 [<0.001, 2.000]	1.000 [<0.001, 1.000]	0.089
NP3RTALL	1.000 [<0.001, 2.000]	1.000 [<0.001, 1.000]	0.266
NP3RTALJ	1.000 [1.000, 2.000]	1.000 [<0.001, 1.000]	0.056
NP3RTCON	1.000 [0.250, 2.000]	1.000 [<0.001, 1.000]	0.029
NP3TOT	47.500 [33.250, 58.750]	34.500 [27.750, 43.250]	0.001

This table summarizes item-level and total scores from the Movement Disorder Society–Unified Parkinson's Disease Rating Scale (MDS-UPDRS). NP1, NP2, and NP3 correspond to MDS-UPDRS Part I (non-motor experiences of daily living), Part II (motor experiences of daily living), and Part III (motor examination), respectively.

Supplemental Table 3 Triangular correlations among FDG-PET regional metabolism, clinical cognitive measures, and oculomotor features

FDG-PET	Clinical variable	Oculomotor feature	P (Clinical–Oculomotor)	P (FDG–Oculomotor)	P (FDG–Clinical)	r (FDG–Clinical)	r (FDG–Oculomotor)
Frontal_Inf_Oper_R	Alternating Attention	AS_CorrectionRate_pct	0.031	0.034	0.030	0.404	0.395
Frontal_Inf_Oper_R	Attention	AS_CorrectionRate_pct	0.003	0.034	0.048	0.370	0.395
Frontal_Inf_Oper_R	Divided Attention	AS_CorrectionRate_pct	0.048	0.034	0.003	0.540	0.395
Frontal_Inf_Oper_R	Executive Function	AS_CorrectionRate_pct	0.002	0.034	0.016	0.444	0.395
Frontal_Inf_Oper_R	EMDCS	AS_CorrectionRate_pct	0.000	0.034	0.002	0.556	0.395
Frontal_Inf_Oper_R	Memory	AS_CorrectionRate_pct	0.002	0.034	0.040	0.383	0.395
Frontal_Inf_Oper_R	Planning Ability	AS_CorrectionRate_pct	0.000	0.034	0.006	0.498	0.395
Frontal_Inf_Oper_R	Processing Speed / Reaction Ability	AS_CorrectionRate_pct	0.012	0.034	0.012	0.458	0.395
Frontal_Inf_Oper_R	Executive Function	AS_MaxAmplitudeDown_deg	0.001	0.027	0.016	0.444	0.410
Frontal_Inf_Oper_R	EMDCS	AS_MaxAmplitudeDown_deg	0.000	0.027	0.002	0.556	0.410
Frontal_Inf_Oper_R	Planning Ability	AS_MaxAmplitudeDown_deg	0.000	0.027	0.006	0.498	0.410
Frontal_Inf_Oper_R	Attention	AS_MaxAmplitudeLeft_deg	0.012	0.028	0.048	0.370	0.407
Frontal_Inf_Oper_R	Divided Attention	AS_MaxAmplitudeLeft_deg	0.035	0.028	0.003	0.540	0.407
Frontal_Inf_Oper_R	Executive Function	AS_MaxAmplitudeLeft_deg	0.001	0.028	0.016	0.444	0.407
Frontal_Inf_Oper_R	EMDCS	AS_MaxAmplitudeLeft_deg	0.000	0.028	0.002	0.556	0.407
Frontal_Inf_Oper_R	Planning Ability	AS_MaxAmplitudeLeft_deg	0.000	0.028	0.006	0.498	0.407
Frontal_Inf_Oper_R	Processing Speed / Reaction Ability	AS_MaxAmplitudeLeft_deg	0.015	0.028	0.012	0.458	0.407
Frontal_Inf_Oper_R	Attention	AS_MaxAmplitudeRight_deg	0.037	0.006	0.048	0.370	0.494
Frontal_Inf_Oper_R	Executive Function	AS_MaxAmplitudeRight_deg	0.000	0.006	0.016	0.444	0.494

Frontal_Inf_Oper_R	EMDCS	AS_MaxAmplitudeRight_deg	0.000	0.006	0.002	0.556	0.494
Frontal_Inf_Oper_R	Memory	AS_MaxAmplitudeRight_deg	0.030	0.006	0.040	0.383	0.494
Frontal_Inf_Oper_R	Planning Ability	AS_MaxAmplitudeRight_deg	0.000	0.006	0.006	0.498	0.494
Frontal_Inf_Oper_R	Attention	AS_UncorrectedErrorRate_pct	0.004	0.033	0.048	0.370	-0.396
Frontal_Inf_Oper_R	Divided Attention	AS_UncorrectedErrorRate_pct	0.048	0.033	0.003	0.540	-0.396
Frontal_Inf_Oper_R	Executive Function	AS_UncorrectedErrorRate_pct	0.000	0.033	0.016	0.444	-0.396
Frontal_Inf_Oper_R	EMDCS	AS_UncorrectedErrorRate_pct	0.000	0.033	0.002	0.556	-0.396
Frontal_Inf_Oper_R	Memory	AS_UncorrectedErrorRate_pct	0.000	0.033	0.040	0.383	-0.396
Frontal_Inf_Oper_R	Planning Ability	AS_UncorrectedErrorRate_pct	0.000	0.033	0.006	0.498	-0.396
Frontal_Inf_Oper_R	Short-term Memory	AS_UncorrectedErrorRate_pct	0.000	0.033	0.050	0.368	-0.396
Frontal_Inf_Oper_R	Planning Ability	GP_SaccadeGain	0.037	0.021	0.006	0.498	0.428
Frontal_Inf_Oper_R	Alternating Attention	OP_CompletionDurationAvg_ms	0.000	0.040	0.030	0.404	-0.384
Frontal_Inf_Oper_R	Attention	OP_CompletionDurationAvg_ms	0.000	0.040	0.048	0.370	-0.384
Frontal_Inf_Oper_R	Divided Attention	OP_CompletionDurationAvg_ms	0.000	0.040	0.003	0.540	-0.384
Frontal_Inf_Oper_R	EMDCS	OP_CompletionDurationAvg_ms	0.006	0.040	0.002	0.556	-0.384
Frontal_Inf_Oper_R	Planning Ability	OP_CompletionDurationAvg_ms	0.017	0.040	0.006	0.498	-0.384
Frontal_Inf_Oper_R	Processing Speed / Reaction Ability	OP_CompletionDurationAvg_ms	0.000	0.040	0.012	0.458	-0.384
Frontal_Inf_Oper_R	Alternating Attention	OP_Latency_ms	0.000	0.045	0.030	0.404	-0.375
Frontal_Inf_Oper_R	Attention	OP_Latency_ms	0.000	0.045	0.048	0.370	-0.375
Frontal_Inf_Oper_R	Divided Attention	OP_Latency_ms	0.000	0.045	0.003	0.540	-0.375
Frontal_Inf_Oper_R	EMDCS	OP_Latency_ms	0.003	0.045	0.002	0.556	-0.375
Frontal_Inf_Oper_R	Planning Ability	OP_Latency_ms	0.026	0.045	0.006	0.498	-0.375
Frontal_Inf_Oper_R	Processing Speed / Reaction Ability	OP_Latency_ms	0.000	0.045	0.012	0.458	-0.375

Frontal_Inf_Orb_R	EMDCS	AS_MaxAmplitudeDown_deg	0.000	0.016	0.022	0.425	0.444
Frontal_Inf_Orb_R	Divided Attention	AS_MaxAmplitudeLeft_deg	0.035	0.007	0.014	0.451	0.492
Frontal_Inf_Orb_R	EMDCS	AS_MaxAmplitudeLeft_deg	0.000	0.007	0.022	0.425	0.492
Frontal_Inf_Orb_R	EMDCS	AS_MaxAmplitudeRight_deg	0.000	0.002	0.022	0.425	0.544
Temporal_Inf_L	Executive Function	AS_CorrectionRate_pct	0.002	0.010	0.016	0.443	0.469
Temporal_Inf_L	EMDCS	AS_CorrectionRate_pct	0.000	0.010	0.005	0.503	0.469
Temporal_Inf_L	Memory	AS_CorrectionRate_pct	0.002	0.010	0.015	0.449	0.469
Temporal_Inf_L	Planning Ability	AS_CorrectionRate_pct	0.000	0.010	0.002	0.549	0.469
Temporal_Inf_L	Executive Function	AS_MaxAmplitudeLeft_deg	0.001	0.006	0.016	0.443	0.494
Temporal_Inf_L	EMDCS	AS_MaxAmplitudeLeft_deg	0.000	0.006	0.005	0.503	0.494
Temporal_Inf_L	Planning Ability	AS_MaxAmplitudeLeft_deg	0.000	0.006	0.002	0.549	0.494
Temporal_Inf_L	Executive Function	AS_MaxAmplitudeRight_deg	0.000	0.007	0.016	0.443	0.492
Temporal_Inf_L	EMDCS	AS_MaxAmplitudeRight_deg	0.000	0.007	0.005	0.503	0.492
Temporal_Inf_L	Memory	AS_MaxAmplitudeRight_deg	0.030	0.007	0.015	0.449	0.492
Temporal_Inf_L	Planning Ability	AS_MaxAmplitudeRight_deg	0.000	0.007	0.002	0.549	0.492
Temporal_Inf_L	Executive Function	AS_MaxAmplitudeUp_deg	0.001	0.007	0.016	0.443	0.486
Temporal_Inf_L	EMDCS	AS_MaxAmplitudeUp_deg	0.005	0.007	0.005	0.503	0.486
Temporal_Inf_L	Memory	AS_MaxAmplitudeUp_deg	0.037	0.007	0.015	0.449	0.486
Temporal_Inf_L	Planning Ability	AS_MaxAmplitudeUp_deg	0.000	0.007	0.002	0.549	0.486
Temporal_Inf_L	Executive Function	AS_UncorrectedErrorRate_pct	0.000	0.011	0.016	0.443	-0.466
Temporal_Inf_L	EMDCS	AS_UncorrectedErrorRate_pct	0.000	0.011	0.005	0.503	-0.466
Temporal_Inf_L	Memory	AS_UncorrectedErrorRate_pct	0.000	0.011	0.015	0.449	-0.466
Temporal_Inf_L	Planning Ability	AS_UncorrectedErrorRate_pct	0.000	0.011	0.002	0.549	-0.466

Temporal_Inf_L	Short-term Memory	AS_UncorrectedErrorRate_pct	0.000	0.011	0.007	0.487	-0.466
Temporal_Inf_L	EMDCS	GP_Latency_ms	0.031	0.041	0.005	0.503	-0.381
Temporal_Inf_L	Planning Ability	GP_Latency_ms	0.023	0.041	0.002	0.549	-0.381
Temporal_Inf_R	Executive Function	AS_Accuracy_pct	0.000	0.012	0.003	0.536	0.462
Temporal_Inf_R	EMDCS	AS_Accuracy_pct	0.000	0.012	0.000	0.654	0.462
Temporal_Inf_R	Inhibitory Control	AS_Accuracy_pct	0.000	0.012	0.039	0.385	0.462
Temporal_Inf_R	Memory	AS_Accuracy_pct	0.000	0.012	0.000	0.648	0.462
Temporal_Inf_R	Planning Ability	AS_Accuracy_pct	0.000	0.012	0.001	0.604	0.462
Temporal_Inf_R	Short-term Memory	AS_Accuracy_pct	0.000	0.012	0.000	0.694	0.462
Temporal_Inf_R	Executive Function	AS_CorrectionRate_pct	0.002	0.010	0.003	0.536	0.470
Temporal_Inf_R	EMDCS	AS_CorrectionRate_pct	0.000	0.010	0.000	0.654	0.470
Temporal_Inf_R	Memory	AS_CorrectionRate_pct	0.002	0.010	0.000	0.648	0.470
Temporal_Inf_R	Planning Ability	AS_CorrectionRate_pct	0.000	0.010	0.001	0.604	0.470
Temporal_Inf_R	Executive Function	AS_MaxAmplitudeLeft_deg	0.001	0.009	0.003	0.536	0.476
Temporal_Inf_R	EMDCS	AS_MaxAmplitudeLeft_deg	0.000	0.009	0.000	0.654	0.476
Temporal_Inf_R	Planning Ability	AS_MaxAmplitudeLeft_deg	0.000	0.009	0.001	0.604	0.476
Temporal_Inf_R	Executive Function	AS_MaxAmplitudeRight_deg	0.000	0.013	0.003	0.536	0.456
Temporal_Inf_R	EMDCS	AS_MaxAmplitudeRight_deg	0.000	0.013	0.000	0.654	0.456
Temporal_Inf_R	Inhibitory Control	AS_MaxAmplitudeRight_deg	0.023	0.013	0.039	0.385	0.456
Temporal_Inf_R	Memory	AS_MaxAmplitudeRight_deg	0.030	0.013	0.000	0.648	0.456
Temporal_Inf_R	Planning Ability	AS_MaxAmplitudeRight_deg	0.000	0.013	0.001	0.604	0.456
Temporal_Inf_R	Executive Function	AS_MaxAmplitudeUp_deg	0.001	0.049	0.003	0.536	0.369
Temporal_Inf_R	EMDCS	AS_MaxAmplitudeUp_deg	0.005	0.049	0.000	0.654	0.369

Temporal_Inf_R	Inhibitory Control	AS_MaxAmplitudeUp_deg	0.032	0.049	0.039	0.385	0.369
Temporal_Inf_R	Memory	AS_MaxAmplitudeUp_deg	0.037	0.049	0.000	0.648	0.369
Temporal_Inf_R	Planning Ability	AS_MaxAmplitudeUp_deg	0.000	0.049	0.001	0.604	0.369
Temporal_Inf_R	Executive Function	AS_UncorrectedErrorRate_pct	0.000	0.003	0.003	0.536	-0.528
Temporal_Inf_R	EMDCS	AS_UncorrectedErrorRate_pct	0.000	0.003	0.000	0.654	-0.528
Temporal_Inf_R	Inhibitory Control	AS_UncorrectedErrorRate_pct	0.002	0.003	0.039	0.385	-0.528
Temporal_Inf_R	Memory	AS_UncorrectedErrorRate_pct	0.000	0.003	0.000	0.648	-0.528
Temporal_Inf_R	Planning Ability	AS_UncorrectedErrorRate_pct	0.000	0.003	0.001	0.604	-0.528
Temporal_Inf_R	Short-term Memory	AS_UncorrectedErrorRate_pct	0.000	0.003	0.000	0.694	-0.528
Temporal_Inf_R	EMDCS	GP_CompletionDurationAvg_ms	0.002	0.036	0.000	0.654	-0.391
Temporal_Inf_R	Memory	GP_CompletionDurationAvg_ms	0.013	0.036	0.000	0.648	-0.391
Temporal_Inf_R	Planning Ability	GP_CompletionDurationAvg_ms	0.018	0.036	0.001	0.604	-0.391
Temporal_Inf_R	Planning Ability	GP_SaccadeGain	0.037	0.027	0.001	0.604	0.411
Temporal_Inf_R	Executive Function	SP_TotalDeviation_gt4deg_deg	0.009	0.048	0.003	0.536	-0.371
Temporal_Inf_R	EMDCS	SP_TotalDeviation_gt4deg_deg	0.025	0.048	0.000	0.654	-0.371
Temporal_Inf_R	Inhibitory Control	SP_TotalDeviation_gt4deg_deg	0.042	0.048	0.039	0.385	-0.371
Temporal_Inf_R	Memory	SP_TotalDeviation_gt4deg_deg	0.014	0.048	0.000	0.648	-0.371
Temporal_Inf_R	Planning Ability	SP_TotalDeviation_gt4deg_deg	0.008	0.048	0.001	0.604	-0.371
Temporal_Inf_R	Short-term Memory	SP_TotalDeviation_gt4deg_deg	0.004	0.048	0.000	0.694	-0.371
Temporal_Inf_R	EMDCS	SP1_TrackingSpeed_dps	0.026	0.018	0.000	0.654	0.437
Temporal_Inf_R	Memory	SP1_TrackingSpeed_dps	0.004	0.018	0.000	0.648	0.437
Temporal_Inf_R	Planning Ability	SP1_TrackingSpeed_dps	0.041	0.018	0.001	0.604	0.437
Temporal_Inf_R	Short-term Memory	SP1_TrackingSpeed_dps	0.022	0.018	0.000	0.694	0.437

Temporal_Inf_R	Executive Function	SP2_OverallAccuracy_pct	0.015	0.026	0.003	0.536	0.414
Temporal_Inf_R	EMDCS	SP2_OverallAccuracy_pct	0.000	0.026	0.000	0.654	0.414
Temporal_Inf_R	Memory	SP2_OverallAccuracy_pct	0.000	0.026	0.000	0.648	0.414
Temporal_Inf_R	Planning Ability	SP2_OverallAccuracy_pct	0.000	0.026	0.001	0.604	0.414
Temporal_Inf_R	Short-term Memory	SP2_OverallAccuracy_pct	0.002	0.026	0.000	0.694	0.414
Temporal_Pole_Sup_R	Attention	AS_CorrectionRate_pct	0.003	0.008	0.007	0.490	0.481
Temporal_Pole_Sup_R	Executive Function	AS_CorrectionRate_pct	0.002	0.008	0.039	0.385	0.481
Temporal_Pole_Sup_R	EMDCS	AS_CorrectionRate_pct	0.000	0.008	0.003	0.535	0.481
Temporal_Pole_Sup_R	Memory	AS_CorrectionRate_pct	0.002	0.008	0.021	0.425	0.481
Temporal_Pole_Sup_R	Planning Ability	AS_CorrectionRate_pct	0.000	0.008	0.002	0.545	0.481
Temporal_Pole_Sup_R	Attention	AS_MaxAmplitudeLeft_deg	0.012	0.024	0.007	0.490	0.418
Temporal_Pole_Sup_R	Executive Function	AS_MaxAmplitudeLeft_deg	0.001	0.024	0.039	0.385	0.418
Temporal_Pole_Sup_R	EMDCS	AS_MaxAmplitudeLeft_deg	0.000	0.024	0.003	0.535	0.418
Temporal_Pole_Sup_R	Planning Ability	AS_MaxAmplitudeLeft_deg	0.000	0.024	0.002	0.545	0.418
Temporal_Pole_Sup_R	Attention	AS_UncorrectedErrorRate_pct	0.004	0.008	0.007	0.490	-0.484
Temporal_Pole_Sup_R	Executive Function	AS_UncorrectedErrorRate_pct	0.000	0.008	0.039	0.385	-0.484
Temporal_Pole_Sup_R	EMDCS	AS_UncorrectedErrorRate_pct	0.000	0.008	0.003	0.535	-0.484
Temporal_Pole_Sup_R	Memory	AS_UncorrectedErrorRate_pct	0.000	0.008	0.021	0.425	-0.484
Temporal_Pole_Sup_R	Planning Ability	AS_UncorrectedErrorRate_pct	0.000	0.008	0.002	0.545	-0.484
Temporal_Pole_Sup_R	Short-term Memory	AS_UncorrectedErrorRate_pct	0.000	0.008	0.012	0.462	-0.484
Temporal_Pole_Sup_R	Attention	GP_Latency_ms	0.000	0.007	0.007	0.490	-0.492
Temporal_Pole_Sup_R	EMDCS	GP_Latency_ms	0.031	0.007	0.003	0.535	-0.492
Temporal_Pole_Sup_R	Planning Ability	GP_Latency_ms	0.023	0.007	0.002	0.545	-0.492

Temporal_Pole_Sup_R	Attention	SP_OverallAccuracy_pct	0.043	0.046	0.007	0.490	0.374
Temporal_Pole_Sup_R	Executive Function	SP_OverallAccuracy_pct	0.041	0.046	0.039	0.385	0.374
Temporal_Pole_Sup_R	Planning Ability	SP_OverallAccuracy_pct	0.044	0.046	0.002	0.545	0.374
Temporal_Pole_Sup_R	Short-term Memory	SP_OverallAccuracy_pct	0.036	0.046	0.012	0.462	0.374
Temporal_Pole_Sup_R	Attention	SP1_OverallAccuracy_pct	0.007	0.008	0.007	0.490	0.480
Temporal_Pole_Sup_R	Executive Function	SP1_OverallAccuracy_pct	0.022	0.008	0.039	0.385	0.480
Temporal_Pole_Sup_R	EMDCS	SP1_OverallAccuracy_pct	0.001	0.008	0.003	0.535	0.480
Temporal_Pole_Sup_R	Memory	SP1_OverallAccuracy_pct	0.006	0.008	0.021	0.425	0.480
Temporal_Pole_Sup_R	Planning Ability	SP1_OverallAccuracy_pct	0.008	0.008	0.002	0.545	0.480
Temporal_Pole_Sup_R	Short-term Memory	SP1_OverallAccuracy_pct	0.015	0.008	0.012	0.462	0.480
Temporal_Pole_Sup_R	EMDCS	SP1_TrackingAcceleration_dps2	0.017	0.011	0.003	0.535	0.463
Temporal_Pole_Sup_R	Memory	SP1_TrackingAcceleration_dps2	0.007	0.011	0.021	0.425	0.463
Temporal_Pole_Sup_R	Short-term Memory	SP1_TrackingAcceleration_dps2	0.034	0.011	0.012	0.462	0.463
Temporal_Pole_Sup_R	EMDCS	SP1_TrackingSpeed_dps	0.026	0.005	0.003	0.535	0.508
Temporal_Pole_Sup_R	Memory	SP1_TrackingSpeed_dps	0.004	0.005	0.021	0.425	0.508
Temporal_Pole_Sup_R	Planning Ability	SP1_TrackingSpeed_dps	0.041	0.005	0.002	0.545	0.508
Temporal_Pole_Sup_R	Short-term Memory	SP1_TrackingSpeed_dps	0.022	0.005	0.012	0.462	0.508
Temporal_Pole_Sup_R	Attention	SP2_OverallAccuracy_pct	0.003	0.004	0.007	0.490	0.515
Temporal_Pole_Sup_R	Executive Function	SP2_OverallAccuracy_pct	0.015	0.004	0.039	0.385	0.515
Temporal_Pole_Sup_R	EMDCS	SP2_OverallAccuracy_pct	0.000	0.004	0.003	0.535	0.515
Temporal_Pole_Sup_R	Memory	SP2_OverallAccuracy_pct	0.000	0.004	0.021	0.425	0.515
Temporal_Pole_Sup_R	Planning Ability	SP2_OverallAccuracy_pct	0.000	0.004	0.002	0.545	0.515

Temporal_Pole_Sup_R	Short-term Memory	SP2_OverallAccuracy_pct	0.002	0.004	0.012	0.462	0.515

EMDCS: the eye-movement–derived cognitive score.

Supplemental Table 4 Cross-validated and test-set performance of machine learning models for PD subtype classification

**Model**	**CV AUC**	**Test AUC**	**Accuracy**	**F1 score**
SVM (RBF)	0.968	0.909	0.806	0.857
LR (L2)	0.966	0.891	0.774	0.837
RF	0.955	0.884	0.774	0.844
XGBoost		0.882	0.774	0.837
AdaBoost	0.952	0.864	0.742	0.826
LightGBM	0.947	0.855	0.742	0.810
LR (L1)	0.976	0.845	0.742	0.810
MLP	0.962	0.845	0.774	0.837

SVM (RBF), support vector machine with a radial basis function kernel; LR (L1/L2), logistic regression with L1 or L2 regularization; RF, random forest; XGBoost, extreme gradient boosting; AdaBoost, adaptive boosting; LightGBM, light gradient boosting machine; MLP, multilayer perceptron.
