# Supplementary material for: Multimodal data-driven eye-movement subtypes and their cerebral glucose metabolic patterns in Parkinson’s disease
Source: Front Aging Neurosci. 2026 Mar 11;18:1794652. doi: 10.3389/fnagi.2026.1794652 (PMC13013447; doi:10.3389/fnagi.2026.1794652)
Supplement: Supplementary file 5 [file Data_Sheet_1.pdf]

Supplemental Figure 1 Unsupervised consensus clustering reveals two oculomotor subtypes in Parkinson's disease

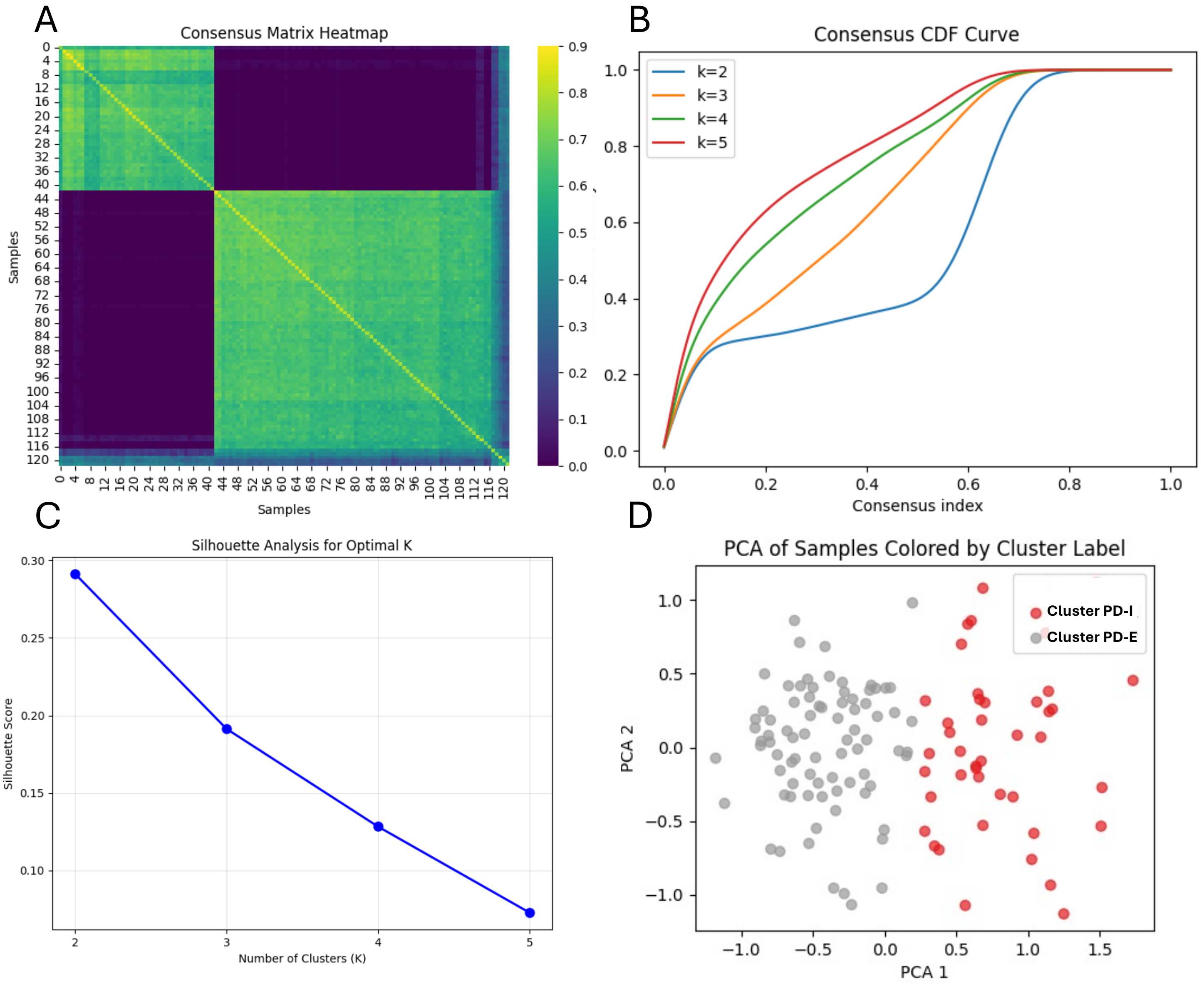

(A) Consensus matrix heatmap for the optimal two-cluster solution (k = 2), demonstrating high within-cluster consensus and low between-cluster similarity.
(B) Cumulative distribution function (CDF) curves for consensus indices across k = 2–5, indicating improved cluster stability at k = 2.
(C) Silhouette analysis across k = 2–5, showing the highest mean silhouette score at k = 2 (mean silhouette = 0.29), supporting optimal between-cluster separation.
(D) Principal component analysis (PCA) of PD samples colored by cluster assignment, illustrating a clear tendency toward separation between the two oculomotor subtypes (PD-I and PD-E).
